# Ketogenic diet attenuates hepatopathy in mouse model of respiratory chain complex III deficiency caused by a *Bcs1l* mutation

**DOI:** 10.1038/s41598-017-01109-4

**Published:** 2017-04-19

**Authors:** Janne Purhonen, Jayasimman Rajendran, Matthias Mörgelin, Kristiina Uusi-Rauva, Shintaro Katayama, Kaarel Krjutskov, Elisabet Einarsdottir, Vidya Velagapudi, Juha Kere, Matti Jauhiainen, Vineta Fellman, Jukka Kallijärvi

**Affiliations:** 1 Folkhälsan Research Center, Helsinki, Finland; 2 Faculty of Medicine, University of Helsinki, Helsinki, Finland; 3 Division of Infection Medicine, Clinical Sciences, Lund University, Lund, Sweden; 4 Department of Biosciences and Nutrition, Karolinska Institutet, Huddinge, Sweden; 5 Competence Centre on Health Technologies, Tartu, Estonia; 6 Metabolomics Unit, Institute for Molecular Medicine, University of Helsinki, Helsinki, Finland; 7 Molecular Neurology Research Program, University of Helsinki, Helsinki, Finland; 8 Department of Genetics and Molecular Medicine, King’s College London, England, UK; 9 National Institute for Health and Welfare, Helsinki, Finland; 10 Department of Clinical Sciences, Lund, Pediatrics, Lund University, Lund, Sweden; 11 Children’s Hospital, Helsinki University Hospital, University of Helsinki, Helsinki, Finland

## Abstract

Mitochondrial disorders are among the most prevalent inborn errors of metabolism but largely lack treatments and have poor outcomes. High-fat, low-carbohydrate ketogenic diets (KDs) have shown beneficial effects in mouse models of mitochondrial myopathies, with induction of mitochondrial biogenesis as the suggested main mechanism. We fed KD to mice with respiratory chain complex III (CIII) deficiency and progressive hepatopathy due to mutated BCS1L, a CIII assembly factor. The mutant mice became persistently ketotic and tolerated the KD for up to 11 weeks. Liver disease progression was attenuated by KD as shown by delayed fibrosis, reduced cell death, inhibition of hepatic progenitor cell response and stellate cell activation, and normalization of liver enzyme activities. Despite no clear signs of increased mitochondrial biogenesis in the liver, CIII assembly and activity were improved and mitochondrial morphology in hepatocytes normalized. Induction of hepatic glutathione transferase genes and elevated total glutathione level were normalized by KD. Histological findings and transcriptome changes indicated modulation of liver macrophage populations by the mutation and the diet. These results reveal a striking beneficial hepatic response to KD in mice with mitochondrial hepatopathy and warrant further investigations of dietary modification in the management of these conditions in patients.

## Introduction

Mitochondrial disorders can manifest as hepatopathy, mainly in infants, with or without other organ manifestations^1^. Only supportive treatments, such as adjustment of nutrition and vitamin supplementation to alleviate the metabolic imbalance, are available for these patients^1, 2^.

Mutations in the *BCS1L* gene, encoding a CIII assembly factor, are the most common diagnosed cause of mitochondrial respiratory chain (RC) CIII (CIII) deficiency^3^. GRACILE syndrome^4^ (fetal growth restriction, aminoaciduria, cholestasis, liver iron overload, lactic acidosis, and early death during infancy) is caused by a homozygous missense mutation (c.232 A > G, p.S78G) in *BCS1L* ^5^. In liver mitochondria of the patients, Rieske iron-sulfur protein (RISP, UQCRFS1) incorporation into CIII is decreased resulting in reduced CIII activity^6^. We have generated a viable mouse model of CIII dysfunction by introducing the *Bcs1l* ^*c.232A>G*^ mutation into the mouse genome^7^. The homozygous mice display growth failure, low blood glucose, high blood lactate and progressive hepatopathy from the fourth week of age, recapitulating many of the main findings in the patients.

Carbohydrate-restricted high-fat ketogenic diets stimulate mitochondrial β-oxidation and ketone body production in the liver^8, 9^. Ketone bodies can be utilized as an alternative energy source by the brain, heart and skeletal muscle. KDs are an established therapy in drug-resistant epilepsies and have been shown to increase the expression of genes related to tricarboxylic acid cycle and RC function, as well as the number of mitochondria in rat hippocampi^8–10^. KDs have been proposed and tested as a therapy for mitochondrial disorders. Krebs *et al*. hypothesized that the course of postweaning-onset mitochondrial cardiomyopathy in *Med30* mutant mice is affected by the considerable decrease in fat intake on shifting from maternal milk (42% fat) to the low-fat regular chow. Indeed, the researchers could extend the survival of the mutant mice by 40% by weaning them to high-fat ketogenic diet^11^. Schiff *et al*. showed that high-fat diet-fed Harlequin mice with CI deficiency exhibit a slower progression of the neurodegenerative phenotype^12^. In a mouse model of late-onset mitochondrial myopathy, KD decreased the amount of cytochrome c oxidase negative muscle fibers and prevented mitochondrial ultrastructural abnormalities in the muscle and normalized metabolic and lipidomic changes^13^. In a subsequent study by this group a modified Atkins diet caused muscle pain in the myopathy patients and selective lysis of abnormal muscle fibers, which was considered eventually beneficial for the muscle function^14^. Moreover, KD has been shown to increase mitochondrial mass in skeletal muscle^13^, heart^15^ and brown adipose tissue^16^.

With the hypothesis that KD may induce mitochondrial biogenesis to compensate reduced RC function, improve energy metabolism and ameliorate disease progression, we performed a dietary intervention to assess the effect of KD on liver disease in the *Bcs1l* mutant model of CIII dysfunction.

## Results

### KD induces persistent ketosis in *Bcs1l* mutant (*Bcs1l*^*G/G*^) mice

KD was fed to cohorts of mutant mice and wild-type (WT) littermates starting from weaning. To monitor ketosis in the feeding cohorts, blood β-hydroxybutyrate levels were measured at the time of sacrificing. WT mice on KD had significantly elevated β-hydroxybutyrate levels at P45, but after that the ketogenic effect decreased (Table 1). In contrast, the KD-fed *Bcs1l* ^*G/G*^ mice were ketotic during the entire intervention. Non-fasting blood glucose level of *Bcs1l* ^*G/G*^ mice was lower than in WT animals but similar on both diets. Plasma creatinine, urea and total protein were normal (Table 1). KD did not influence weight gain in either genotype (Supplementary Fig. 1). *Bcs1l* ^*G/G*^ mice tolerated KD from weaning for up to two and half months and thereafter showed behavioral decline suggesting adverse effect of the diet (data not shown).

**Table 1:** Biochemical parameters in whole blood and plasma, mean ± SD (n).

	Age (d)	WT		*Bcs1l* ^G/G^	
		CD	KD	CD	KD
**Whole blood**					
Glucose (mmol/L)	45	8.1 ± 1.3 (7)	8.3 ± 1.3 (7)	5.6 ± 2.0^†††^ (9)	5.0 ± 1.9 (7)
	95	8.9 ± 1.6 (8)	10.3 ± 2.0 (9)	4.7 ± 1.1^†††^ (8)	3.2 ± 1.1 (6)
Lactate (mmol/L)	45	3.2 ± 1.9 (9)	2.7 ± 2.4 (7)	5.0 ± 4.6 (9)	3.1 ± 2.8 (8)
	95	8.8 ± 2.0 (8)	4.3 ± 2.3 (9)	6.5 ± 3.6 (8)	8.1 ± 3.4 (6)
Lactate to glucose ratio	45	0.36 ± 0.22 (7)	0.36 ± 0.32 (6)	0.90 ± 0.84 (8)	0.67 ± 0.68 (7)
	95	0.8 ± 0.3 (8)	0.4 ± 0.3 (9)	1.3 ± 0.6^†^ (8)	2.3 ± 0.9^‡‡^ (6)
β-hydroxybutyrate (mmol/L)	45	0.36 ± 0.3 (6)	2.0 ± 0.6*** (3)	0.58 ± 0.2 (4)	4.8 ± 1.1^‡‡‡^ (3)
	95	0.58 ± 0.2 (8)	0.67 ± 0.2 (9)	0.87 ± 0.4 (8)	4.9 ± 2.0^‡‡^ (6)
**Plasma**	all 95				
Alanine aminotransferase (U/L)		42 ± 8 (5)	88 ± 67 (5)	260 ± 75^†††^ (5)	170 ± 71 (5)
Alkaline phosphatase (U/L)		57 ± 19 (5)	30 ± 23 (5)	190 ± 56^†††^ (5)	80 ± 40^‡^ (5)
Creatinine (µmol/L)		16 ± 4 (5)	17 ± 3 (5)	20 ± 3 (5)	28 ± 6 (5)
Urea (mmol/L)		8.3 ± 1.3 (5)	6.5 ± 0.9 (5)	8.5 ± 0,6 (5)	6.8 ± 2.4 (5)
Uric acid (µmol/L)		75 ± 33 (5)	51 ± 20 (5)	140 ± 35^†^ (5)	160 ± 110 (5)
Total protein (g/L)		49 ± 2 (5)	53 ± 4 (5)	46 ± 5 (5)	52 ± 5 (5)

Non-fasting blood samples were used for the measurements. Significances *p* < 0.05, < 0.01 and < 0.001 are indicated for comparisons between WT on CD vs WT on KD (*, ** and ***), WT on CD vs *Bcs1l* ^*G/G*^ on CD (^†^, ^††^, ^†††^) and *Bcs1l* ^*G/G*^ on CD vs *Bcs1l* ^*G/G*^ *on* KD (^‡^, ^‡‡^, ^‡‡‡^), respectively. n numbers in brackets.

### KD delays liver fibrosis and inhibits stellate cell activation and hepatic progenitor cell response in *Bcs1l*^*G/G*^ mice

**Figure 1:**
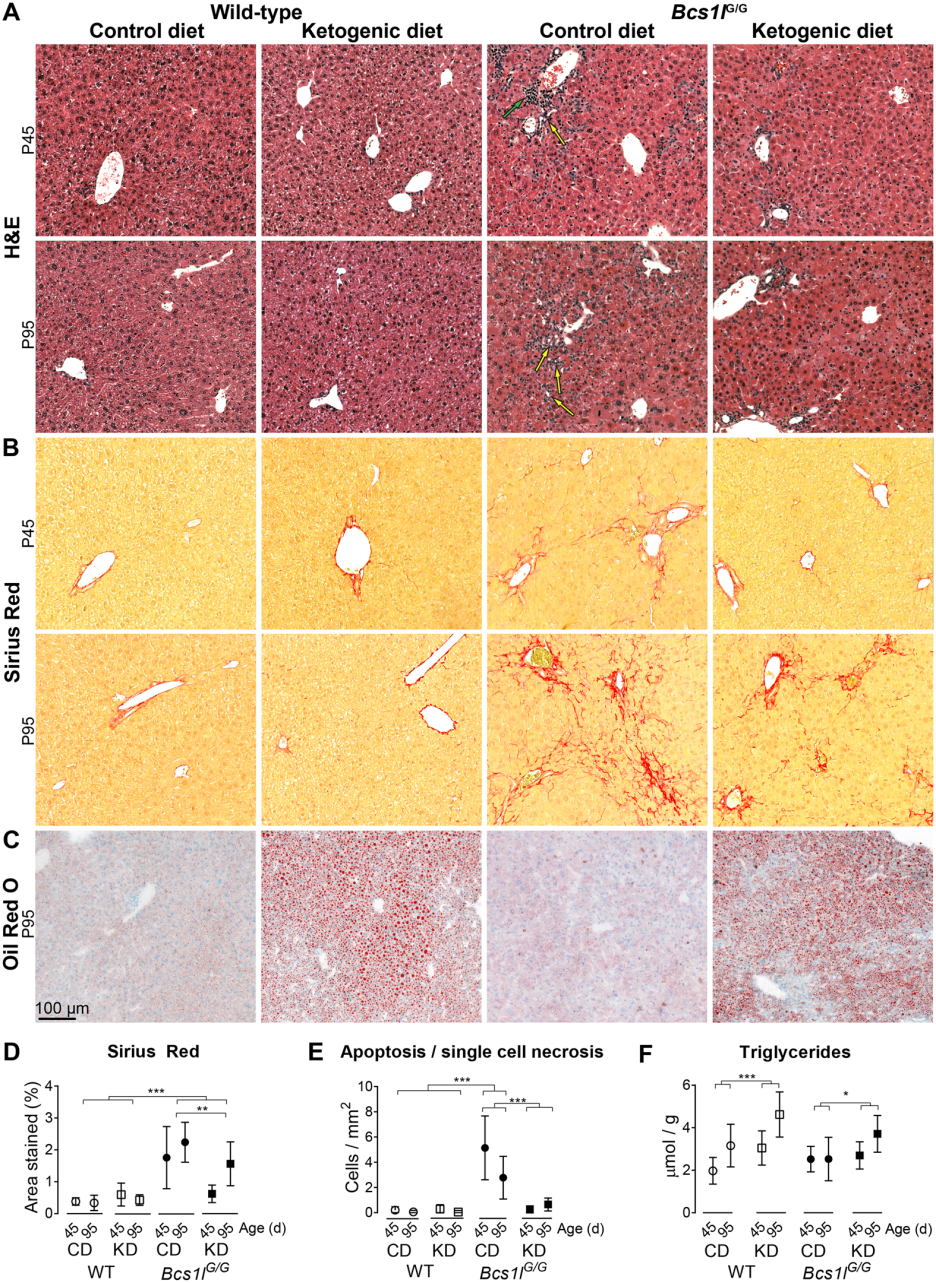
Ketogenic diet modulates hepatopathy progression in *Bcs1l* ^*G/G*^ mice. (**A**) Representative H&E stained liver sections showing ductular reactions (yellow arrows) and portal inflammation (green arrow) in the *Bcs1l* ^*G/G*^ mice on control diet (CD). The ketogenic diet (KD) normalized the expansion of portal areas at postnatal day 45 (P45). A partial amelioration was also observed at postnatal day 95 (P95). For higher-magnification images and their description see Supplementary Fig. 3. (**B**) Sirius Red staining of liver collagen showing incipient fibrosis at P45 and marked periportal and sinusoidal fibrosis at P95 in the *Bcs1l* ^*G/G*^ mice on CD. (**C**) Oil Red O staining of liver cryosections to show neutral lipid accumulation. (**D**) Quantification of fibrosis from Sirius Red-stained liver sections showing delayed fibrosis in the *Bcs1l* ^*G/G*^ mice on KD. n = 5–8/group for *Bcs1l* ^*G/G*^ mice (both time points) and 3–4/group for WT mice at P45 and 8–9 /group at P95, respectively. **p < 0.01, ***p < 0.001 (two-way ANOVA with age group as a factor to be controlled) (**E**) Count of apoptotic and necrotic cells in H&E-stained liver sections. ***p < 0.001 (Mann-Whitney U-test) (**F**) Liver triglyceride assay. ***p = 0.0001 for the diet effect, * *p* = 0.44 for diet*age interaction (two-way ANOVA). n = 7–13/group. The error bars stand for standard deviation. Abbreviations: WT, wild-type mice; *Bcs1l* ^*G/G*^, mice homozygous for the *Bcs1l* ^*c.232A>G*^ knock-in mutation.

**Figure 2:**
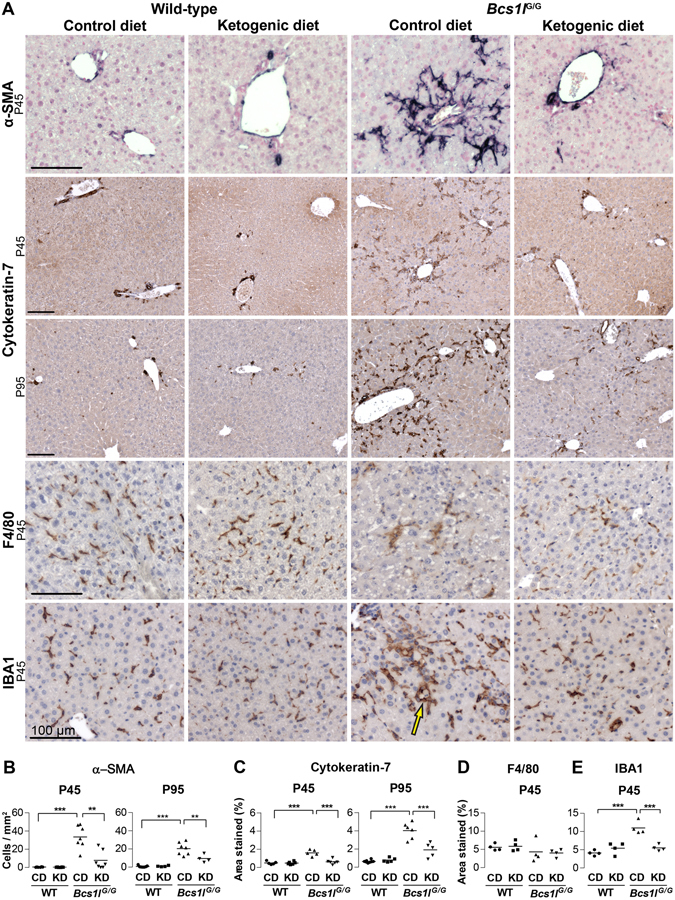
Ketogenic diet modulates activation of hepatic progenitor cells, stellate cells and liver macrophages in *Bcs1l* ^*G/G*^ mice. (**A**) Immunostainings of liver sections for hepatic progenitor cell and bile duct marker cytokeratin-7; activated stellate cell marker α-smooth muscle actin (α-SMA) and macrophage markers F4/80 and IBA1. (**A,B**) In wild-type livers α-SMA staining (dark blue) is seen only in the smooth muscle layer of arterioles but in the *Bcs1l* ^*G/G*^ mice on CD staining appears in numerous activated spindle-like stellate cells. In the *Bcs1l* ^*G/G*^ mice on KD the stellate cell α-SMA signal is diminished. (**A, C**) In wild-type livers cytokeratin-7 staining (dark brown) is only seen in bile ducts whereas in *Bcs1l* ^*G/G*^ livers the staining is present in numerous cells in the liver parenchyma. The abnormal staining is almost completely absent in the *Bcs1l* ^*G/G*^ mice on KD at P45. (**A,D,E**) F4/80 and IBA1 stainings (dark brown) show normal Kupffer cells in wild-type livers. IBA1 staining is increased in *Bcs1l* ^*G/G*^ livers but normalized by KD. Characteristic IBA1-positive macrophages surrounding a hepatocyte (crown-like structure, arrow) in *Bcs1l* ^*G/G*^ mice. All scale bars present 100 μm. **p < 0.01; ***p < 0.001.

In WT mice, KD had a minimal effect on liver histology (Fig. 1A) but caused a modest increase in liver triglyceride amount (Fig. 1C,F) and reduced liver glycogen content (Supplementary Fig. 2). Non-esterified fatty acid concentration was similar in all groups (Supplementary Fig. 3). *Bcs1l* ^*G/G*^ mice on control diet (CD) had early-stage hepatopathy characterized by expansion of portal triads with incipient fibrosis at P45 (Fig. 1A). Marked hepatocyte hypertrophy was observed centrivenously and to some degree throughout the lobules (Supplementary Fig. 4B,C). Apoptotic cells, single necrotic cells (Fig. 1E) and mitotic figures were frequent. Staining for the mitotic cell marker Ki-67 confirmed a proliferative response at P45, which tended to resolve by P95 (Supplementary Fig. 5). Collagen staining showed periportal fibrosis at P45 (Fig. 1B) and at P95 prominent pericellular fibrosis was also observed. Occasionally fibrotic septa connected two or more portal triads. Highly increased α-smooth muscle actin (α-SMA) immunostaining indicated stellate cell activation (Fig. 2A,B).

KD modulated progression of the hepatopathy in several ways (Figs 1 and 2): it reduced the number of apoptotic and necrotic cells, prevented expansion of portal areas and delayed progression of fibrosis (Fig. 1) and diminished liver glycogen content (Supplementary Fig. 2). The expansion of portal areas resembled hepatic progenitor cell response (ductular reactions and oval cell hyperplasia)^17^ (Fig. 1A and Supplementary Fig. 4D). Accordingly, immunostaining revealed aberrant expression of cytokeratin-7, a marker of bile ducts and hepatic progenitor cells (oval cells), mainly periportally but also across the parenchyma (Fig. 2A,C). The aberrant cytokeratin-7 expression was completely normalized by KD at P45 and attenuated at P95 (Fig. 2A,C). Also plasma alanine aminotransferase and alkaline phosphatase activities were reduced by KD (Table 1). Further details of the liver histology are shown in Supplementary Fig. 4.

### KD normalizes the ultrastructure of hepatocyte mitochondria and improves CIII assembly and activity in *Bcs1l*^*G/G*^ livers

**Figure 3:**
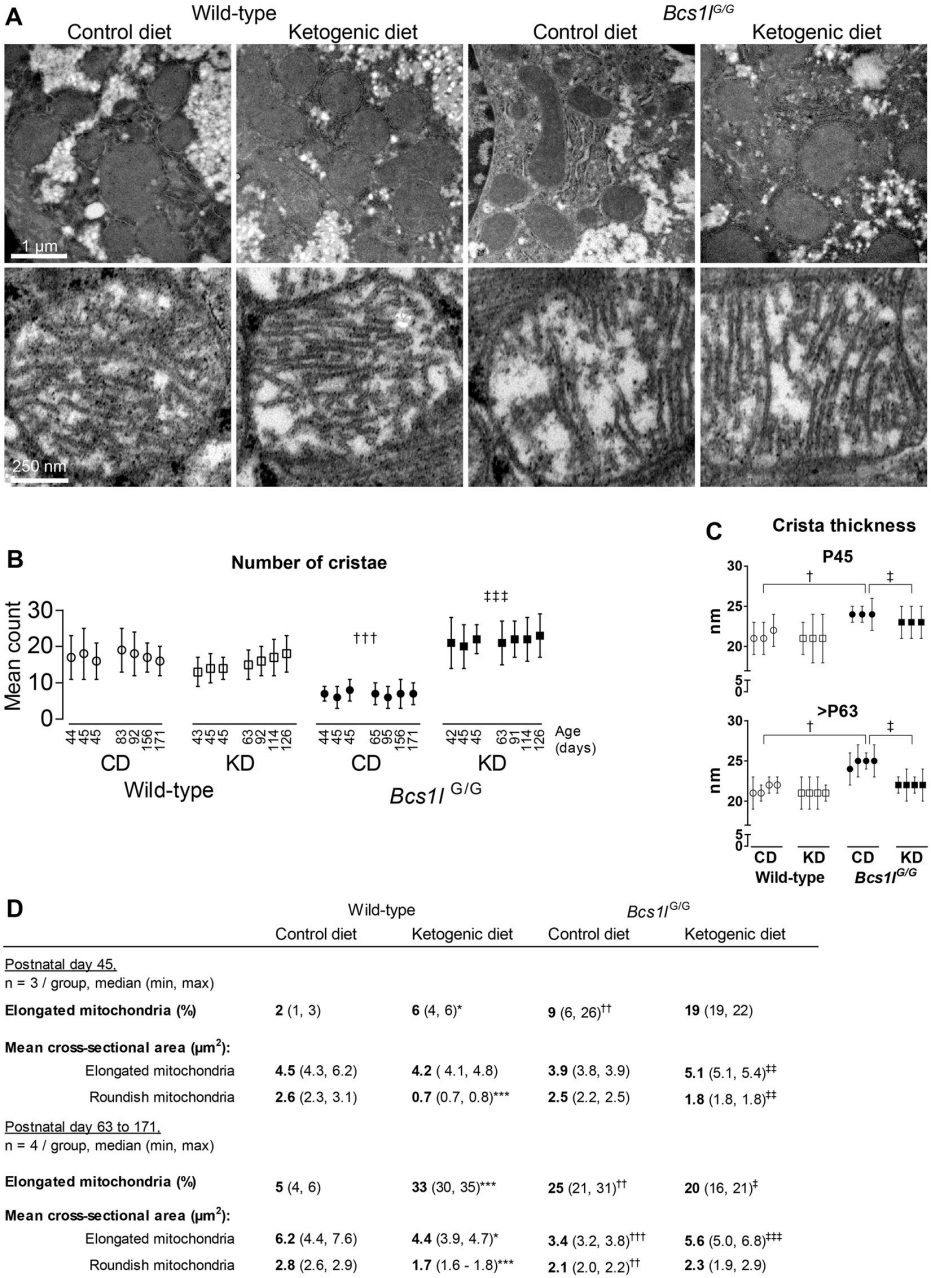
Ultrastructure of hepatocyte mitochondria is modulated by the *Bcs1l* mutation and the diet. (**A**) Upper row of representative electron micrographs shows general mitochondrial morphology in hepatocytes and lower panel typical cristae morphology and number of cristae in wild-type and *Bcs1l* ^*G/G*^ mice on CD and KD. (**B-C**) Mean mitochondrion cristae number and thickness. The data points represent five hundred mitochondria per mouse (mean ± SD). (**D**) Quantification of elongated and roundish mitochondria. Statistics: 1-way ANOVA followed by planned comparisons for all parameters except for cristae thickness for which Kruskal-Wallis test followed by Mann-Whitney U-tests was used. *p* < 0.05, *p* < 0.01, *p* < 0.001 (*, ** and *** vs WT CD), (^†^, ^††^ and ^†††^ vs WT CD), (^‡^, ^‡‡^ and ^‡‡‡^ vs *Bcs1l* ^*G/G*^ CD).

Electron microscopy showed that in hepatocyte mitochondria of *Bcs1l* ^*G/G*^ mice the number of cristae was reduced and thickness increased as compared to WT mice (Fig. 3A–C). Both parameters were normalized in the KD-fed mice at all time points (P45-P171). The proportion of elongated mitochondria was increased by the mutation, KD and age of the mice (Fig. 3D). The elongated mitochondria in KD-fed *Bcs1l* ^*G/G*^ mice had also a larger cross-sectional area than the mitochondria in the mutant mice on CD (Fig. 3D).

**Figure 4:**
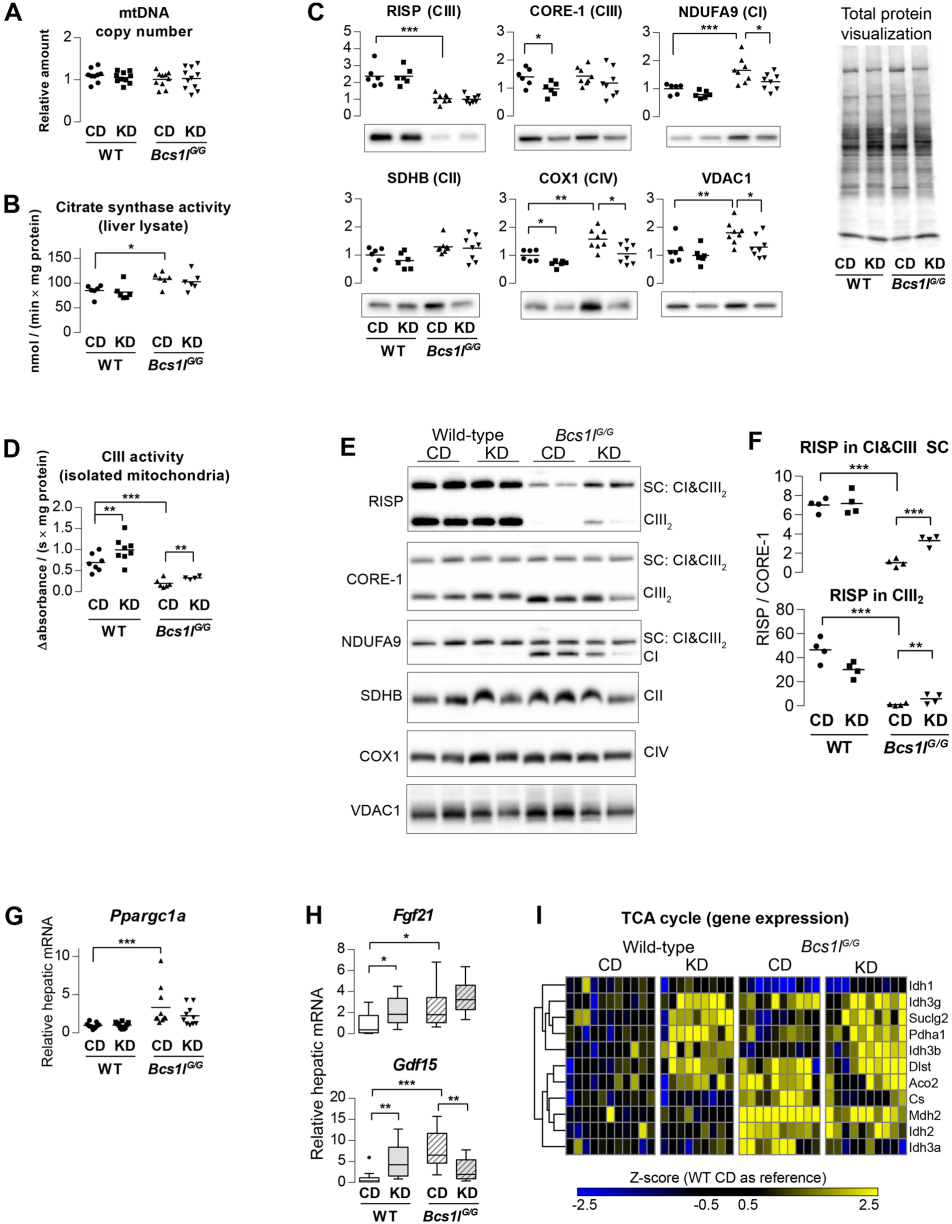
Ketogenic diet improves CIII assembly and activity but does not increase mitochondrial mass in *Bcs1l* ^*G/G*^ livers. (**A**) Liver mitochondrial DNA (mtDNA) relative to nuclear DNA. (**B**) Citrate synthase activity in liver lysate as a marker of mitochondria content. (**C**) Western blot analysis of RC proteins and VDAC1 (PORIN) in liver lysates. The data were normalized to total protein transferred to membrane as quantified from total protein staining. (**D**) Spectrophotometric analysis of CIII activity in isolated liver mitochondria. (**E**) Blue native polyacrylamide gel electrophoresis of digitonin-solubilized mitochondria and subsequent Western blot and analysis of intact RC complexes and supercomplexes (SC). **(F**) Quantification of RISP amount in CIII dimer (CIII_2_) and in supercomplex containing CI and CIII_2_. The band intensities were normalized to CIII subunit CORE-1. (**G**) Relative expression of *Ppargc1a*, a master regulator of mitochondrial biogenesis. (**H**) Hepatic expression (RNA-seq) of two proposed mitochondrial disease markers Fibroblast growth factor 21 (*Fgf21*) and Growth/differentiation factor 15 (*Gdf15*). n = 9–11/group. The box plot whiskers represent minimum and maximum values within the 1.5 interquartile range. (**I**) Differentially expressed genes in liver transcriptome with annotation (GO:0006099) to tricarboxylic acid cycle (TCA cycle). CIII activity and BNGE were done from the P95 panel, all other measurements from the P45 panel. *p < 0.05,**p < 0.01; ***p < 0.001. Western blot images were cropped for clarity in C and E. The full images are shown in Supplementary Figs. 15 and 16. Abbreviations: official gene symbols were used; CI, respiratory chain complex I; CD, control diet; KD, ketogenic diet; NDUFA9, NADH:ubiquinone oxidoreductase subunit A9 ; SDHB, succinate dehydrogenase complex iron sulfur subunit B; CORE-1, Ubiquinol-cytochrome-c reductase complex core protein 1; RISP, Rieske iron-sulfur protein; COX1, Cytochrome c oxidase subunit 1.

Of the two main markers of mitochondrial mass, mtDNA copy number was unchanged between the groups (Fig. 4A), whereas citrate synthase (CS) activity was increased in *Bcs1l* ^*G/G*^ livers but not affected by KD (Fig. 4B). Western blot analysis of *Bcs1l* ^*G/G*^ liver lysates revealed, as expected^7^, reduced level of RISP and a similar amount of the earlier assembled CIII subunit CORE-1 as compared to WT lysates (Fig. 4C). The amount of neither CIII subunit was affected by KD in total liver lysates. VDAC1, a mitochondrial protein not part of the RC, was increased in liver lysates of the *Bcs1l* ^*G/G*^ mice on CD, as were also subunits of CI (NDUFA9) and CIV (COX1, cytochrome c oxidase I, MT-CO1). The level of these proteins was normalized by KD (Fig. 4C). CIII activity in isolated *Bcs1l* ^*G/G*^ liver mitochondria was reduced by approximately 70% on normal diet. KD increased CIII activity approximately 50% in both genotypes (Fig. 4D). Blue native gel electrophoresis (BNGE) and subsequent immunoblotting of intact complexes in isolated liver mitochondria showed that the amount of RISP in free CIII dimer and, in particular, in the fully assembled CI-CIII supercomplex was significantly increased in the mutant mice on KD as compared to CD (Fig. 4E). Free CI was abundant only in the mutant mice but normal levels of supercomplex-bound CI were observed. No gross differences were found in the amount of CII and CIV between the groups (Fig. 4E).

In skeletal muscle, CS activity was significantly increased by KD in both genotypes and RISP amount reduced in the mutant mice, but none of the RC complex subunits analyzed were changed (Supplementary Fig. 6). In brown adipose tissue, RISP level was reduced in the mutant mice but neither CS, RC subunits nor uncoupling protein 1 (UCP1) were changed (Supplementary Fig. 7).

### Liver transcriptome changes reflect regenerative response, activated lysosomal function and upregulation of tricarboxylic acid cycle in early symptomatic (P40-45) *Bcs1l*^*G/G*^ mice

**Figure 5:**
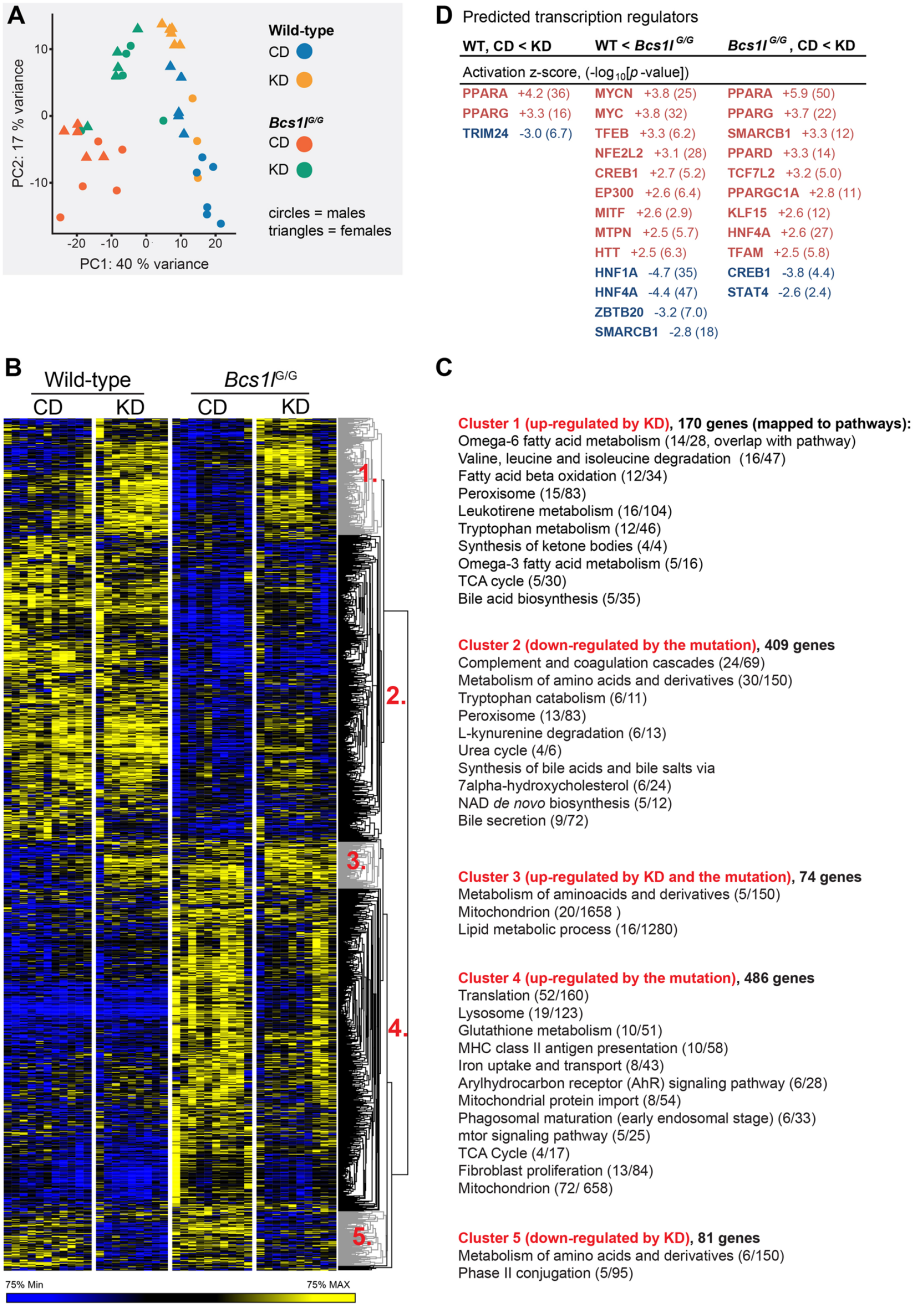
Liver transcriptome is distinctly altered by the *Bcs1l* mutation and the diet. (**A**) Principal component analysis of 500 most variable transcripts shows separation of WT and mutant mice (PC1) and partial separation of dietary groups and gender (PC2). (**B**) Heat map of differentially expressed genes. Nineteen genes had significant gender-genotype interaction (not shown in the heatmap) (**C**) A pathway analysis of the gene clusters. **(D**) The most significant transcriptional regulators predicted to drive the observed gene expression changes.

Principal component analysis (Fig. 5A), profiles of differentially expressed genes (Fig. 5B) and pathway analyses (Supplementary Table 1) demonstrated that of the examined conditions the mutation had the greatest effect on the liver transcriptome (1200 differentially expressed genes). KD changed the expression of approximately 260 genes in WT mice and 460 genes in *Bcs1l* ^*G/G*^ mice. The largest block of up-regulated genes in *Bcs1l* ^*G/G*^ mice was related to translation and ribosomes (Fig. 5B,C and Supplementary Table 1), likely reflecting the regenerative response. Lysosome-related genes were also overrepresented among the up-regulated genes in *Bcs1l* ^*G/G*^ livers. The genes with the highest differential expression are listed in Supplementary Table 2.

The levels of transcripts encoding RC components were mostly unaltered between the groups (Supplementary Fig. 8). An exception was *Uqrcb* encoding a CIII subunit which was up-regulated 3.7-fold by KD in both genotypes (*p* < 0.0001). Of mitochondria-related genes, the majority of tricarboxylic acid cycle genes were induced by both the mutation and the diet (Fig. 4I). The hepatic expression of two circulating mitochondrial disease markers, *Fgf21* ^18^ and *Gdf15* ^19^ was up-regulated in *Bcs1l* ^*G/G*^ mice and *Gdf15* expression was normalized by KD (Fig. 4H). Upstream regulatory analysis predicted that KD activated peroxisome proliferator-activated receptors (PPAR-α,-γ and -α), TFAM and PGC-1α and inhibited CREB, all transcriptional regulators of mitochondrial biogenesis (Fig. 5D). The observed mRNA level of *Ppargc1a* (*PGC-1α*) was increased in the *Bcs1l* ^*G/G*^ mice but not affected by the diet (Fig. 4G). Expression of a recently identified mitophagy and mitochondrial mass regulator *Bnip3* ^20^ was increased 1.4-fold (*p* = 0.02) and 1.8-fold (*p* < 0.001) by KD in WT mice and *Bcs1l* ^*G/G*^ mice, respectively. *Bcs1l* ^*G/G*^ mice on CD had decreased (-1.6-fold) expression of *Bnip3* and related autophagy regulators, Atg3 (−1.8-fold) and Atg5 (−1.5-fold) compared to WT mice on CD (all *p* < 0.001).

### Amino acid and lipid metabolism are altered at transcriptome and metabolite levels in *Bcs1l*^*G/G*^ mice

The expression of multiple amino acid starvation response genes (i.e. *Atf3*, *Atf4*, *Ddit3, Trib3*, *Asns* and *Txnip*) was up-regulated in *Bcs1l* ^*G/G*^ mice (Supplementary Fig. 9). Amino acid metabolism-related genes were enriched in all comparisons (Fig. 5B,C and Supplementary Fig. 10A). Targeted plasma metabolomics revealed elevated concentrations of eight amino acids in the *Bcs1l* ^*G/G*^ mice (Supplementary Fig. 10B). In line with a probable increase in protein catabolism, gene expression pathway analyses predicted altered urea cycle activity in *Bcs1l* ^*G/G*^ livers (Fig. 5B,C), and, accordingly, the plasma level of arginine was decreased and ornithine and citrulline increased (Supplementary Fig. 10B).

Gene expression pathway analyses further suggested that hepatic lipid metabolism was profoundly altered by the mutation and the diet (Fig. 5B,C, Supplementary Fig. 11A and Supplementary Table 1). The expression of genes related to ketone body synthesis, bile acid biosynthesis and ω-3 and ω-6 fatty acid metabolism were up-regulated by KD (Fig. 5B,C). Several bile acid biosynthesis-related genes were down-regulated in *Bcs1l* ^*G/G*^ mice (Fig. 5B,C). Metabolomics analysis showed increased plasma concentrations of cholic acid and chenodeoxycholic (Supplementary Fig. 11B). Also, the concentrations of plasma carnitine and short-chain acylcarnitines were decreased by the mutation and KD further indicating altered lipid metabolism (Supplementary Fig. 11B).

### KD modulates altered hepatic macrophage populations in *Bcs1l*^*G/G*^ mice

In H&E-stained liver sections of *Bcs1l* ^*G/G*^ mice, cells with brownish cytoplasm and morphologically resembling macrophages were observed (Supplementary Fig. 12). These cells were PAS-diastase-positive and auto-fluorescent (Supplementary Fig. 12), suggesting that they were ceroid- or lipofuscin-laden macrophages. The number of these cells was decreased by KD. Immunostaining for macrophage/Kupffer cell markers showed similar number of hepatic macrophages with both F4/80 and IBA1 antibodies in WT mice (Fig. 2D,E). F4/80 is an established marker for tissue resident macrophages but some infiltrating monocyte-derived macrophages are negative or only weakly immunopositive for F4/80^21, 22^. In the *Bcs1l* ^*G/G*^ livers F4/80 staining was reduced suggesting either loss of this marker or Kupffer cell depletion. However, the number of IBA1-expressing macrophages was increased in *Bcs1l* ^*G/G*^ livers and normalized by KD at P45 (Fig. 2A,E). In *Bcs1l* ^*G/G*^ livers the macrophages were larger and protruded out of sinusoids, or multiple macrophages surrounded a hepatocyte (Fig. 2A). Neutrophil count was marginally increased in the mutant livers (Supplementary Fig. 13).

**Figure 6:**
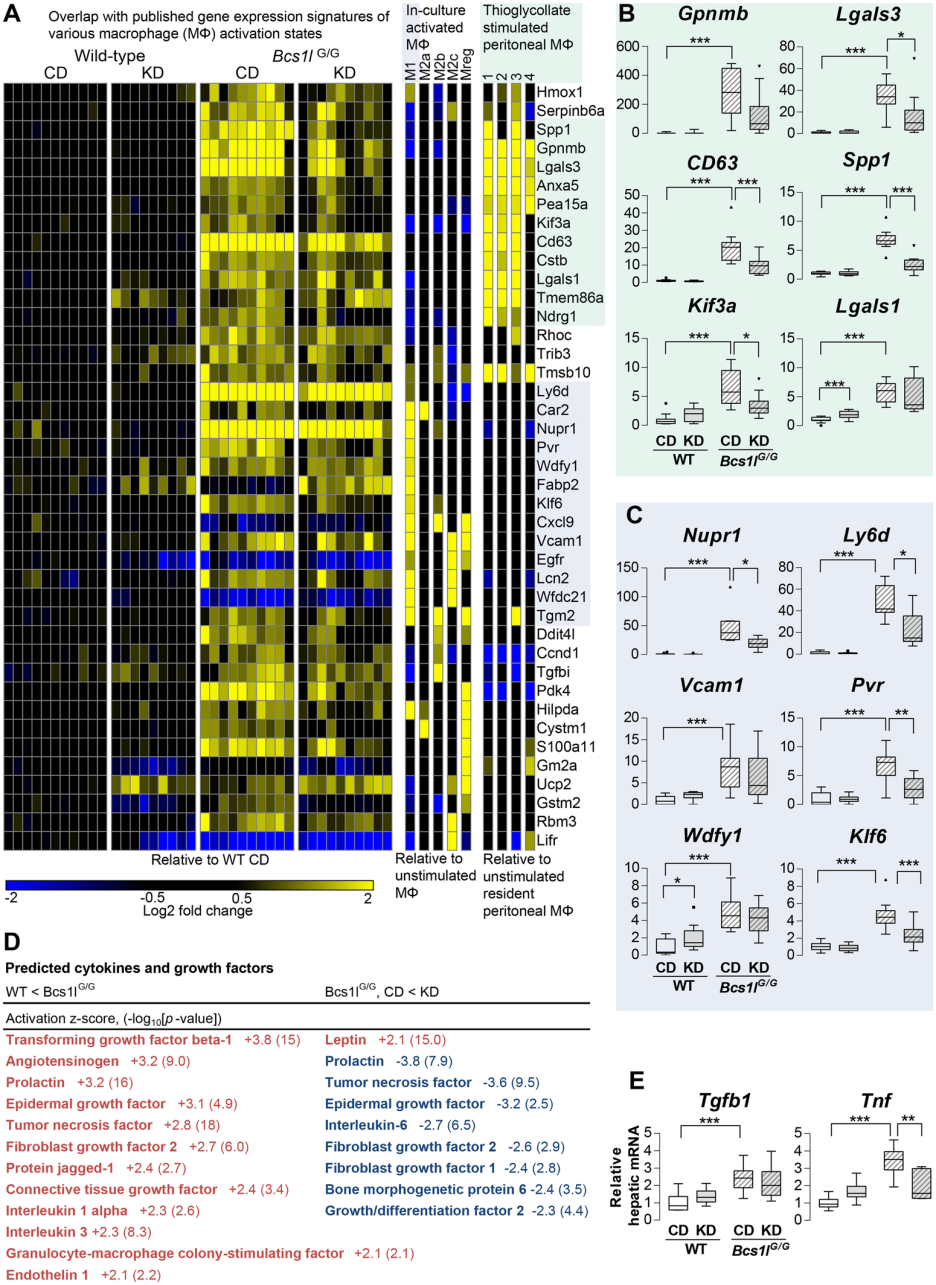
Ketogenic diet modulates macrophage (MΦ) polarization-related gene expression signatures in *Bcs1l* ^*G/G*^ mice. (**A**) Liver transcriptomic data overlapping with published MΦ gene expression signatures. Expression profiles of in-culture stimulated mouse MΦ (induced from bone marrrow derived monocytes) were obtained from Gene expression omnibus (E-GEOD-32690)^62^. The macrophage populations are named as in the original work: M1, IFN-γ + LPS; M2a, IL-4; M2b, IFN-γ + complexed Ig; M2c, Dexamethasone; Mreg, IFN-γ. Expression profiles of four thioglycollate stimulated mouse peritoneal MΦ populations (flow cytometry sorted for CD115^pos^, MHC-II, F4/80, SiglecF^neg^ and CD11c^pos^) were extracted from microarray dataset GSE1590. Liver transcriptome data was filtered for *q* < 0.1 and DESeq2 adjusted fold change > 3 in any of the three pair-wise comparison (WT on CD vs KD, WT on CD vs *Bcs1l* ^*G/G*^ on CD and *Bcs1l* ^*G/G*^ on CD vs KD). MΦ datasets were filtered with the same criteria except that only up-regulated genes were included. (**B**) The most highly up-regulated genes overlapping with the profile of thioglycollate stimulated peritoneal MΦ. (**C**) The most highly up-regulated genes overlapping with the profile of M1-type MΦ. (**D**) Ingenuity Upstream Regulator Analysis for cytokines and growth factors predicted to drive observed gene expression changes. (**E**) Hepatic expression of transforming growth factor beta-1 (*Tgfb1*) and tumor necrosis factor (*Tnf*) as measured by qPCR. In the box plots n = 9–11/group except for *Tnf* for which n = 6–8/group. The box plot whiskers represent minimum and maximum values within the 1.5 interquartile range. *p < 0.05, **p < 0.01, ***p < 0.001.

Gene expression pathway analyses (Supplementary Table 1) indicated changes in four inflammation related pathways (Reactome database) in the *Bcs1l/* ^*G/G*^ mice: formation of fibrin clot, infectious disease, MHC class II antigen presentation and phagosomal maturation (*p* < 0.0003 for all). Furthermore, one fifth of genes that were up-regulated more than 3-fold in *Bcs1l/* ^*G/G*^ mice overlapped with genes that play a role in innate immune response (InnateDB^23^). For example the macrophage marker gene *Cd68* was up-regulated 8.5-fold (*p* < 0.0001) in the *Bcs1l/* ^*G/G*^ mice and downregulated 2.4-fold (*p* = 0.0046) by KD. Comparison to various published macrophage gene expression profiles showed that most of the highly up-regulated genes in *Bcs1l/* ^*G/G*^ livers were linked to activated macrophages (Fig. 6A). KD normalized the majority of these gene expression changes towards WT levels.

The expression of the central inflammation and fibrosis-related cytokines, tumor necrosis factor (*Tnf*) and transforming growth factor beta-1 (*Tgfb1*) was upregulated in *Bcs1l/* ^*G/G*^ livers, which was in line with the activation predicted from transcriptomics data (Fig. 6D,E). Of these, *Tnf* expression was significantly normalized by KD (Fig. 6D,E).

### KD normalizes perturbed hepatic glutathione metabolism in *Bcs1l*^*G/G*^ mice

**Figure 7:**
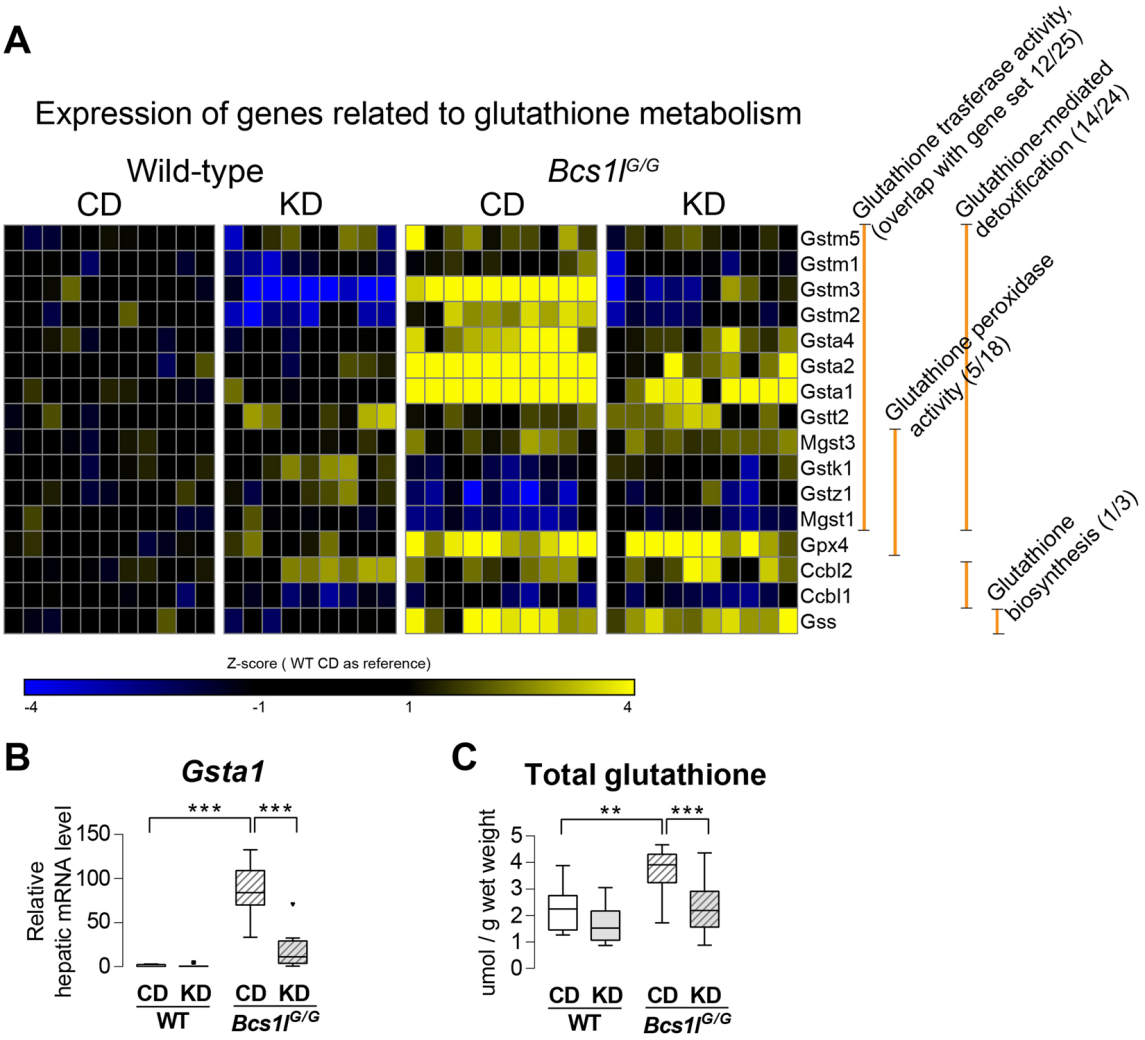
Liver transcriptome analysis reveals perturbed glutathione metabolism in *Bcs1l* ^*G/G*^ mice and its normalization by ketogenic diet. (**A**) Differentially expressed genes with annotation to glutathione metabolism and their subannotation to different branches of glutathione metabolism [glutathione transferase activity GO:0004364, glutathione peroxidase activity GO:0004602, glutathione-mediated detoxification (source: HumanCyc), glutathione biosynthesis (HumanCyc)]. (**B**) Hepatic mRNA level (RNA-seq) of *Gsta1*. (**C**) Hepatic total glutathione level. The results were normalized to total protein concentration and approximate glutathione content per wet weight is presented. The box plot whiskers represent minimum and maximum values within the 1.5 interquartile range. **p < 0.01; ***p < 0.001. All results are from the postnatal day 45 age.

The expression of nine out of seventeen canonical glutathione S-transferase genes detected was up-regulated in *Bcs1l* ^*G/G*^ mice and normalized toward WT levels on KD (Fig. 7A). The most highly up-regulated glutathione transferase gene was *Gsta1* (87-fold, *p* < 0.0001, Fig. 7B), which was down-regulated 4.4-fold by KD (*p* = 0.005). The expression of *Glutathione synthetase* (*Gss*) was increased 2-fold in *Bcs1l* ^*G/G*^ mutants (*p* < 0.001*)*. In line with the gene expression data, total liver glutathione was increased in the *Bcs1l/* ^*G/G*^ mice at P45 and normalized to WT levels on KD (Fig. 7C).

## Discussion

Low-carbohydrate, high-fat diets are an established treatment in drug-resistant epilepsies. Studies in animal models have suggested that modulation of mitochondria-related functions, including reduced reactive oxygen species production, promotion of mitochondrial biogenesis and stimulation of glutathione biosynthesis contribute to their beneficial effects in the brain^8–10^. Here, we demonstrate that KD ameliorated hepatopathy in CIII deficient *Bcs1l* mutant mice at the level of liver histology and liver enzymes, mitochondrial structure and function, and gene expression. Marked stellate cell activation and hepatic progenitor cell proliferation^24^ indicate severe early liver injury in these mice despite relatively modest other histological changes. KD feeding reduced fibrosis, number of apoptotic/necrotic cells, and attenuated stellate cell and hepatic progenitor cell activation, suggesting that it either reduced the initial insult (CIII deficiency), affected fibrogenic processes in the liver or induced protective mechanisms (e.g. detoxification).

We found structural aberrations in mitochondria, the most striking of which was reduced number and increased thickness of cristae, in hepatocytes of *Bcs1l* ^*G/G*^ mice, and these alterations were prevented by KD. In a previous study, KD was shown to normalize overall mitochondrial structure in skeletal muscle in a mouse model of mitochondrial myopathy^13^. Mitochondrial cristae are structurally highly dynamic bioenergetic compartments and their shape is linked to RC function^25, 26^. The reduced number and increased thickness of cristae in hepatocytes of *Bcs1l* ^*G/G*^ mice on CD might be related to disrupted RC complex organization^25^. Starvation has been shown to cause elongation of mitochondria and both starvation and nonglycolytic substrates cause cristae remodeling, including increase in cristae number and reduced width in cultured cells. This process is thought to increase the efficiency of ATP production^27–29^. It is tempting to speculate that the carbohydrate-restricted KD might have caused a similar starvation-like or substrate utilization-dependent mitochondrial adaptation in our mouse model, leading to normalization of cristae number and morphology.

To investigate if the normalized mitochondrial morphology reflected improved mitochondrial function, we measured CIII activity and assessed organization of respiratory chain complexes and supercomplexes in liver mitochondria. Indeed, CIII activity was increased by half in the mice on KD, which may have been sufficient to ameliorate the liver injury due to CIII dysfunction. Previously, a short term (<24 h) KD administration was also shown to increase combined CII and CIII activity in brain cortical mitochondria in a rat mode of traumatic brain injury^30^. Typical markers of mitochondrial mass and biogenesis, such as *Ppargc1a* expression and citrate synthase activity were modestly increased in the mutant mice but not affected by the diet. MtDNA copy number was similar in all groups and none of the RC subunits analyzed were increased by KD. To the contrary, some RC subunits that were increased by the mutation were decreased to WT levels on KD. Transcriptome changes predicted activation of key regulators of mitochondrial biogenesis, PPAR-α,-γ and -α, PGC-1α and TFAM by KD, but apparently this transcriptional response did not result in increased mitochondrial mass. Ketone bodies have been shown to restore complex I stability and activity in a complex I deficient cell line^31^. Therefore, we assessed RC complex and supercomplex amount in liver mitochondria of the mice using BNGE. Despite that KD did not increase total amount of RISP in whole tissue lysate, more fully assembled CIII was found in isolated mitochondria from *Bcs1l* ^*G/G*^ mice on KD than on CD. This finding suggests improved CIII assembly or stability and offers an explanation for the increased CIII activity on KD. Dietary fat composition has been shown to affect phospholipid composition of mitochondrial membranes^32^ and affect respiratory chain supercomplex assembly and function^33, 34^. Our KD contained a high proportion of polyunsaturated long-chain fatty acids, which may have had an effect on mitochondrial membrane composition and, subsequently, on CIII and supercomplex composition in our model.

Previously, a ketogenic diet, with a more extreme protein restriction than our formulation (see later discussion), decreased liver mitochondrial mass likely by up-regulating gene and protein expression of starvation induced mitophagy factor, BNIP3^35^. We observed that KD increased hepatic *Bnip3* expression in both genotypes. The *Bcs1l* ^*G/G*^ mice on CD had decreased *Bnip3* transcript levels compared to WT mice along with reduced mRNA levels of the autophagy regulators *Atg3* and *Atg5* ^36^. Taken together, our results indicate that the increased CIII activity and the beneficial effect of KD were not due to increased liver mitochondrial mass but likely to other adaptive mitochondrial responses such as improved mitochondrial quality control via autophagy and improved maintenance of cristae structure and RC function, including improved CIII assembly and activity.

Hepatic immune cells, including Kupffer cells and infiltrating monocyte-derived macrophages are key pro- and anti-fibrotic regulators^37^. The *Bcs1l* ^*G/G*^ mice had substantially altered liver macrophage populations as shown by immunostainings and macrophage-related gene expression patterns, and these were clearly modulated by the diet. KD also reduced the number of ceroid-lipofuscin-laden macrophages in *Bcs1l* ^*G/G*^ livers, likely reflecting a reduced need for clearance of cellular debris due to reduced cell death. A reduced number of F4/80 positive macrophages, but increased total macrophage count, has been shown previously in the acute phase of liver injury^21, 22^. These studies also identified the weakly F4/80-immunopositive subsets as infiltrating monocyte-derived macrophages. In contrast, the number of IBA1, CD68 and galectin-3 positive macrophages increases upon liver injury^38, 39^. We observed depletion of F4/80-expressing macrophages in *Bcs1l* ^*G/G*^ livers but an increased number of IBA1-positive macrophages, the latter of which was reduced on KD. Expression of galectin-3 (*Lgals3*) and *Cd68* was highly induced in *Bcs1l* ^*G/G*^ on CD and significantly less so on KD. Of interest, macrophage polarization and their inflammatory properties can be modified by mitochondrial function^40^. The M1, or classically activated, subset of macrophages is mainly relying on anaerobic glycolysis whereas the M2, or alternative activated subset, is dependent on oxidative phosphorylation. The M2-like subsets are particularly important in tissue repair and resolution of inflammation^40^. Interestingly, mice with global CI subunit *Ndufs4* knock-out have systemic inflammation and osteopetrosis due to shift in macrophage polarization and osteoclast differentiation^41^.

In our model, components of the KD may have affected liver disease progression via mechanisms unrelated to energy metabolism. Docosahexanoid acid, a long-chain polyunsaturated n-3 fatty acid, can suppress mitochondrial membrane transition pore opening, a key event leading to apoptosis^32^. Indeed, we found significantly less dying hepatocytes in *Bcs1l* ^*G/G*^ mice on KD. Certain long-chain fatty acids and their derivatives are ligands for PPAR-α and -γ^42^. In our KD-fed mice, the expression of several PPAR-α- and PPAR-γ-regulated genes was altered in the liver transcriptome, suggesting that diet-dependent modulation of ligand binding to PPAR-α and -γ may have played a role. Other putative mediators are HCAR2, a receptor for the major ketone body β-hydroxybyturate, expressed in white adipose tissue and some immune cells including macrophages^43, 44^ and FFAR4 (free fatty acid receptor 4), the activation of which modulates macrophage polarization^34, 35^. FFAR4 is activated by long-chain fatty acids and our KD formulation provided 2.8-fold more α-linolenic acid (C18:3 n-3) relative to energy content than the CD. The putative role of these receptors in mediating the effect of the KD should be further studied by administering ketone esters or the respective receptor agonists.

Induction of liver detoxification genes was minimal in *Bcs1l* ^*G/G*^ mice except for glutathione S-transferase (GST) genes. GSTs detoxify a variety of compounds, including oxidative stress-induced metabolites such as 4-hydroxynonenal^45^. Some GSTs also possess peroxidase activity, and as such are antioxidant enzymes. In addition, the majority of GST genes contain antioxidant response element in their promoter region and are regulatory targets of NRF2, the major oxidative stress responsive transcription factor. The expression of several GST genes was highly induced in the liver of *Bcs1l* mice and this induction was partially reversed by KD feeding. Interestingly, the gene expression profile of GSTs in *Bcs1l* ^*G/G*^ mice showed similar induction of cytoplasmic GST genes as observed in the mouse liver when NRF2 pathway is over activated^46^. As for oxidative stress, we found up-regulated expression of three genes encoding antioxidant enzymes (Gpx3, Gpx4 and Nqo1) in the liver of *Bcs1l* ^*G/G*^ mice as well as of glutathione synthetase. Intriguingly, catalase expression was downregulated 1.8 fold (data not shown). KD had no effect on the expression of these genes. In line with the gene expression data liver total glutathione content was increased in *Bcs1l* mice on CD but reduced to WT levels on KD. We interpret these results so that there was reduced need for detoxification and less cellular glutathione synthesized when the *Bcs1l* ^*G/G*^ mice were on KD.

A small retrospective study found KD treatment effective and relatively safe in respiratory chain defects related childhood epilepsy^9^. However, a KD intervention trial in mitochondrial myopathy patients had to be prematurely discontinued due to muscle pain and selective muscle fiber damage^47^. We found that *Bcs1l* ^*G/G*^ mice showed eventual intolerance to the diet manifesting as more rapid behavioral decline after eleven weeks of feeding. Depleted glycogen stores might have led to an exacerbated energy crisis. However, blood glucose and lactate values at sacrificing time were not significantly different on the two diets. Common rodent KD formulations contain only 5E% protein and provide inadequate choline and methionine intake^48, 49^. These KDs cause robust ketosis but also hinder growth of the mice and in some studies have caused adverse liver effects including steatosis^48–50^. KD formulations with higher protein content are nutritionally more suitable but reduce the ketogenic effect in rodents^51^. In line with this we observed only transient ketosis in WT mice while the *Bcs1l* mice remained ketotic during the entire intervention. The KD-fed mice had similar weight gain as those mice on CD and no overt signs of protein malnutrition (weight loss or reduced plasma total protein), which is in line with findings from studies using KD with similar protein content (~9E%)^48, 52^. Liver fat content was increased on KD in both genotypes but according to triglyceride measurements this increase did not fulfill the criteria of steatosis (50 mg/g, 57µMol/g triglycerides)^53^. Our previous study showed a starvation-like condition and high levels amino acids in liver of *Bcs1l* mice at end-stage disease^54^. In this study, metabolomics analysis showed similar elevation of plasma amino acids in *Bcs1l* ^*G/G*^ mice on both diets, which suggests that the probable catabolic state was not exacerbated by KD.

The present study shows that KD may have therapeutic potential in mitochondrial hepatopathies, currently incurable disorders. Further studies in animal models, and eventually in patients, should be designed to elaborate the effect of ketogenic diets on mitochondrial function and inflammatory processes in the context of respiratory chain deficiency.

## Methods

### Animal experiments

Congenic *Bcs1l* ^*c*.232A>G^ mice^7, 55^ were maintained by crossing heterozygotes to wild-type C57BL/6JCrl mice. The survival of the *Bcs1l* ^*c*.232A>G^ homozygous (*Bcs1l* ^*G/G*^) mice in this background is on average 200 days. The animal ethics committee of the State Provincial Office of Southern Finland approved the animal experiments (permit numbers ESAVI-2010-07284/Ym-23 and ESAVI/6142/04.10.07/2014) and the experiments were carried out in accordance with the permits, Laboratory Animal Centre of the University of Helsinki practices and the Federation of Laboratory Animal Science Associations (FELASA) guidelines.

Litters were randomized to CD or KD. The CD (Teklad 2018, Harlan) was a cereal and soy-based rodent chow providing 18% of energy (E%) as fat, 58E% as carbohydrates and 24E% as protein. The KD (TD.96355, Harlan) was based on vegetable shortening, corn oil and casein, providing 90.5E% fat (1/3 as polyunsaturated fatty acids; 30.8 E% linoleic acid and 2.5E% α-linolenic acid), 0.4E% carbohydrates and 9.1 E% protein. Both diets provided approximately the same amount of choline (~8.5 mg/100 kJ) and methionine (~30 mg/100 kJ). The KD-chow was introduced into the cages from one week of age. From weaning on, the pups randomized to KD received *ad libitum* KD only. The CD and KD groups were sacrificed at postnatal age 40–45 days (which we refer as P45-group, n = 11–14/group) or P90-P114 (P95-group, n = 8–9/group). One cohort was followed until deterioration (P114-P173, n = 4/group). Both genders were used and for each homozygote a wild-type or heterozygote control was included. These control animals are referred to as wild-type (WT). The heterozygous mice are phenotypically wild-type^7^. Significant gender differences were not observed if not otherwise stated.

### Sample collection

Blood glucose, lactate and ketone concentrations were measured from tail tip blood under brief isoflurane anesthesia. For other samples, the mice were sacrificed with carbon dioxide inhalation and cervical dislocation or, for blood collection via cardiac puncture, injected with a lethal dose of pentobarbital. The mice were not fasted prior to sample collection due to the hypoglycemia associated with the phenotype of the homozygotes. All samples were collected at the same time of the day during the light period of the mice. Blood was collected by cardiac puncture into Li-heparin tubes and plasma was separated and stored at −80 °C. Tissues were collected into 10% histology-grade formalin or snap frozen in liquid nitrogen and stored at −80 °C.

### Histology and immunostaining

Liver sections were processed and stained with standard methods to assess general histology (hematoxylin-eosin), glycogen content (periodic acid-Shiff, PAS), neutral fat content (Oil Red O), fibrosis (Sirius Red) and ultrastructure (electron microscopy). Primary antibodies used in immunohistochemical staining are listed in Supplementary Table 3. Vectastain Elite peroxidase reagents (Vector Laboratories) were used for detection. Apoptotic and necrotic cells were counted from H&E-stained sections based on characteristic morphological features such as condensed hypereosinophilic cytoplasm, cell swelling and condensed, fragmented or dissolved nucleus according to published guidelines^56^. All quantifications were performed in random-blind fashion. Technical details of the image analyses are described in Supplementary Materials and Methods.

### Electron microscopy

Approximately 1 mm^3^ pieces of liver were fixed in 1.5% glutaraldehyde, 1.5% paraformaldehyde in 0.1 M Sörensen buffer pH 7.2, and processed for electron microscopy according to standard methods. Area, cristae number and thickness, and the shape of hepatocyte mitochondria were determined manually from images in Adobe Photoshop CS5. Cross sectional area was determined by manually selecting the longest and shortest axis of each mitochondria. Approximately 100 different hepatocytes were chosen at random locations and layers throughout at least 10 different specimen preparations. About five randomly chosen mitochondria per hepatocyte were measured, giving a total of approximately 500 mitochondria per independent sample.

### Clinical chemistry and plasma metabolomics

Clinical chemistry parameters were measured from plasma samples using Siemens ADVIA1650 analyzer. Targeted plasma metabolomics (101 metabolites) was performed using an ACQUITY UPLC-MS/MS system as previously described^57^.

### Lipid analyses

Liver triglycerides were measured as enzymatic release of glycerol after methanol-chloroform extraction of lipids as previously described^58^. The same lipid extracts were used to measure non-esterified fatty acids by an enzymatic colorimetric assay (Roche Diagnostics).

### Protein analysis

Western blot analyses were used to quantify the amount of proteins in tissue lysates. The antibodies used are shown in Supplementary Table 3 and technical details in Supplementary Materials and Methods. For the quantifications, 28 independent samples were run in a random order. For representative blots, pooled lysates of 6–8 samples were used. The data were normalized to total protein transferred onto membrane as visualized and quantified by Stain-free imaging technology (Bio-Rad). Digitonin-solubilized respiratory chain complexes and supercomplexes were assessed with BNGE and immunoblotting as described earlier^55^ except that Bio-Rad tank electroblotting apparatus and 1-hour transfer time was used. The BNGE analysis was performed twice on four mice per group with consistent results.

### Enzyme activity assays

CIII activity in isolated mitochondria was measured with decylubiquinol as substrate and cytochrome c as final electron acceptor in a spectrophotometric assay^7^. Citrate synthase activity was measured with a commercial kit (Sigma-Aldrich Cat.no CS0720).

### RNA sequencing (RNA-seq) and quantitative PCR (qPCR)

For library preparation, sequencing and data pre-processing we followed a published protocol^59^ except that 10 ng total RNA from liver tissue rather than single cells were used. Differential gene expression analysis and read count normalization were performed with DESeq2 package for R^60^. For gene set enrichment analyses and heat maps, the gene lists were filtered for absolute DESeq2 shrunken fold change of more than 1.4 and false discovery rate corrected *p*-value less than 0.1 if not otherwise stated. The enriched gene sets were retrieved from ConsensusPathDB^61^. Ingenuity Upstream Regulator Analysis (Qiagen) was used to predict activated or inhibited transcription regulators, cytokines and growth factors. Public microarray gene expression data sets were obtained from the Gene Expression Omnibus and analyzed using GEO2R (both by NCBI) (E-GEOD-32690)^62^ or from the ImmGen database^63^ as a pre-analyzed data set (GSE1590)^63^. The expression of 45 genes was verified by qPCR (Supplementary Fig. 12 and Supplementary Materials and Methods).

### Mitochondrial DNA (mtDNA) copy number quantitation

mtDNA copy number in total liver genomic DNA was determined by measuring the relative amount of a mitochondrial (*MT-Rnr1*) and a nuclear gene (*Actb*) with qPCR.

Liver total glutathione was assessed with GSH-Glo™ Glutathione kit (Promega).

### Statistics (excluding transcriptomics and metabolomics)

As a main strategy to test whether the groups differed, one-way ANOVA was performed. Significant ANOVA results were followed by three planned comparisons: 1) WT on CD vs WT on KD, 2) WT on CD vs *Bcs1l* ^*G/G*^ on CD and 3) *Bcs1l* ^*G/G*^ on CD vs *Bcs1l* ^*G/G*^ on KD. If the normality assumption of ANOVA was violated, Kruskal-Wallis test and Mann-Whitney U-tests were used instead. Two-way ANOVA was used where age of the mice needed to be included in the analysis. The Bonferroni correction was applied to the two-way ANOVA results.

Further information is provided in Supplementary Materials and Methods.

## Electronic supplementary material

Supplementary material
