# Supplementary material for: Ketogenic diet attenuates hepatopathy in mouse model of respiratory chain complex III deficiency caused by a Bcs1l mutation
Source: Sci Rep. 2017 Apr 19;7:957. doi: 10.1038/s41598-017-01109-4 (PMC5430426; doi:10.1038/s41598-017-01109-4)
Supplement: Supplementary file 1 — Supplementary material [file 41598_2017_1109_MOESM1_ESM.pdf]

Ketogenic diet attenuates hepatopathy in mouse model of respiratory chain complex III deficiency caused by a *Bcs1l* mutation

Janne Purhonen, Jayasimman Rajendran, Matthias Mörgelin, Kristiina Uusi-Rauva, Shintaro Katayama, Kaarel Krjutskov, Elisabet Einarsdottir, Vidya Velagapudi, Juha Kere, Matti Jauhiainen, Vineta Fellman, Jukka Kallijärvi

Supplementary materials and methods

Animal housing and disease scoring. The mice were housed in individually ventilated cages at 22-23°C and kept on a 12 h light/dark cycle at the animal facilities of University of Helsinki, Finland. Disease progression was assessed using a sickness score (0 = null; 1 = moderate and 2 = severe) based on six behavioral characteristics (waddling gait, reduced curiosity, immobility, appearance of kyphosis, loss of balance, and reduced grip strength). If the total score reached >7/12, the mice were considered as having end-stage disease and were sacrificed.

Blood glucose, lactate and ketone measurements. Blood glucose, lactate and ketone concentrations were measured with Freestyle Lite and FreeStyle Precision (Abbott Diabetes Care), and Lactate Pro (Arkray Inc.) meters and sticks, respectively.

Electron microscopy. The ultrathin liver sections were examined in a FEI CM 100 BioTWIN transmission electron microscope (Philips) at 60 kV. Images were recorded with a side-mounted Olympus Veleta camera (Olympus Corp.) with a resolution of 2048 x 2048 pixels (2k x 2K). Five hundred mitochondria were analyzed per animal.

Image analyses. Area stained by Sirius Red and cytokeratin-7 was quantified using color range and threshold tools of Photoshop CS4 (Adobe Systems Inc.). Five to six random 100x-fields (approximately 8 mm^2), excluding larger veins and areas not representing liver parenchyma, were quantified. For Sirius Red a batch-dependent single threshold was used. For cytokeratin-7 automatic threshold was used based on median intensity (background intensity) and a correction factor. Area stained with F4/80 and IBA1 was quantified from six 200x-fields (2.1 mm^2) using color deconvolution and automatic threshold plugins of Fiji package of ImageJ[1]. Activated stellate cells (α-SMA staining) and neutrophils (Ly6G staining) were manually counted from area covering 3.5 mm^2 and 0.9 mm^2, respectively. Automatic cell counting with Fiji ImageJ using color deconvolution, threshold and Analyze Particles plugins was used for lipofuscin positive cells and Ki-67 positive cells (area quantified 2.1 mm^2 and 4.5 mm^2, respectively). Relative liver glycogen content was obtained by measuring image intensity difference between PAS stained liver section with and without diastase treatment.

Protein concentration measurements. The protein concentrations were measured with the Bradford reagent (Bio-Rad) with a modification suggested by Zor and Selinger[2]. Detergent interference with the assay was overcome by diluting the samples 1:10 in water and by taking a ratio between 595 and 466 nm absorbance instead of conventional single read-out at 595 nm. Bovine serum albumin was used as a standard.

Immunoblotting. Snap-frozen tissue samples were homogenized in cold lysis buffer (50 mM Tris-HCL pH 7.4, 1% Triton X-100, 0.5% Na-deoxycholate, 0.1% SDS, 25 mM NaF, 1 mM Na_3VO_4, and 1 mM EGTA) containing protease inhibitor mix (Roche) and cleared by centrifugation (15 000g, +4°C). Equal amounts (10-15 µg) of reduced and denatured protein were run on Stain-Free[tm] Tris-glycine 4 - 20 % gradient gels (Bio-Rad) in a random order. The resolved proteins were then transferred onto

PVDF membrane using Trans-Blot Turbo semi-dry system (Bio-Rad). Amount of protein transferred onto membranes was visualized, imaged and quantified utilizing Bio-Rad Stain-Free^{tm} system and later used for data normalization. Then membranes were probed with the antibodies described in Supplementary Table 3. Horseradish peroxidase-conjugated secondary antibodies (Cell Signaling Technology) and enhanced chemiluminescence (Pierce ECL plus, Thermo Scientific) were used for detection. The luminescence was recorded with a Chemidoc MP CCD imager (Bio-Rad) and quantification performed with Image-lab software form the same manufacturer.

Blue Native Gel Electrophoresis (BNGE). Frozen mitochondrial pellets were resuspended in phosphate buffered saline supplemented with Complete Mini Protease Inhibitor (Roche Diagnostics Scandinavia AB, Stockholm, Sweden), and the protein concentrations were estimated by measuring A_{280} with a spectrophotometer (NanoDrop, Thermo Scientific). The mitochondria were sedimented by centrifugation at 5000g for 5 minutes and subsequently dissolved to a concentration of 5 mg protein/ml in MB2 buffer (1.75 M aminocaproic acid, 75 mM Bis-Tris, pH 7.0, 2 mM EDTA, pH 8.0). Mitochondrial membrane proteins were solubilized by incubation in 0.8% digitonin (Sigma-Aldrich) for 5 min on ice. Samples were centrifuged at 13000g for 30 minutes, the supernatant was collected and the protein concentration estimated as before. Finally, SBG [(750 mM aminocaproic acid, 5% Serva Blue G (SERVA Electrophoresis GmbH, Heidelberg, Germany)] was added to a final concentration of 4.5%. Approximately 10µg of total protein was run on Novex NativePAGE^{TM} 4-16% Bis-Tris (1mm x 10 well) gels (Life Technologies) and transferred to polyvinylidene fluoride membrane using tank electroblotting apparatus (Bio-Rad) and tris-glycine transfer buffer containing 20% MeOH and 0.05% SDS.

RNA extraction. RNA was extracted from 20 - 25 mg liver pieces with NucleoSpin-RNA kit from Macherey-Nagel with on-column DNase treatment. RNA integrity was assessed with Agilent 2100 Bioanalyzer. Total RNA concentration was measured with Qubit 2.0 fluorometer (Thermo Scientific).

Transcriptomics data analyses. The DESeq2 package[3], used for differential gene expression analysis, was run through the Chipster-interface (v.3.8.0)[4]. RNA extraction batch information was included in the analysis as a factor to be controlled. The heat maps were produced using GENE-E software by the Broad Institute (*http://www.broadinstitute.org/cancer/software/GENE-E/*).

Quantitative PCR (qPCR). Total RNA was reverse transcribed with RevertAid H Minus kit (Thermo Scientific) using random hexamers. SYBR green chemistry (DyNAmo Flash SYBR Green qPCR kit, Thermo Scientific), Bio-Rad CFX96 Real-Time PCR Detection System and Bio-Rad CFX Manager (v.3.1) software were employed to perform the qPCR. Primer sequences are available upon request. PCR efficiency was assessed from the slope of dilution series or directly from the amplification data with LinRegPCR software[5]. Five reference genes (*Actb*, *Ywhaz*, *Srp72*, *Gak* and *Vps4a*) were tested, and from these *Gak* and *Vps4a* were chosen for normalization of the expression data. The expression values were calculated using the $\Delta\Delta Cq$ method for efficiency corrected Cq values.

Statistics. The normality of ANOVA residuals was tested by the Shapiro-Wilk test and inspecting the histograms. Equality of variances was tested with the Levene test. For analyses not part of the main strategy, the Bonferroni correction was applied. The analyses were performed using SPSS statistics 22 (IBM Corp.).

Supplementary references

1. Schindelin J. et al. Fiji: an open source platform for biological-image analysis. Nat Meth **9**, 676-682 (2012).

2. Zor T, Selinger Z. Linearization of the Bradford protein assay increases its sensitivity: theoretical and experimental studies. Anal. Biochem **236**, 302-308 (1996).

3. Love MI, Huber W, Anders S. Moderated estimation of fold change and dispersion for RNA-seq data with DESeq2. Genome Biol **15**, 1-21 (2014).

4. Kallio MA. et al. Chipster: user-friendly analysis software for microarray and other high-throughput data. BMC Genomics **12**, 1-14 (2016).

5. Ruijter JM. et al. Amplification efficiency: linking baseline and bias in the analysis of quantitative PCR data. Nucleic Acids Res. **37**, e45 (2009).

6. Tang X. et al. Comprehensive Profiling of Amino Acid Response Uncovers Unique Methionine-Deprived Response Dependent on Intact Creatine Biosynthesis. PLoS Genet **11**, e1005158 (2015).

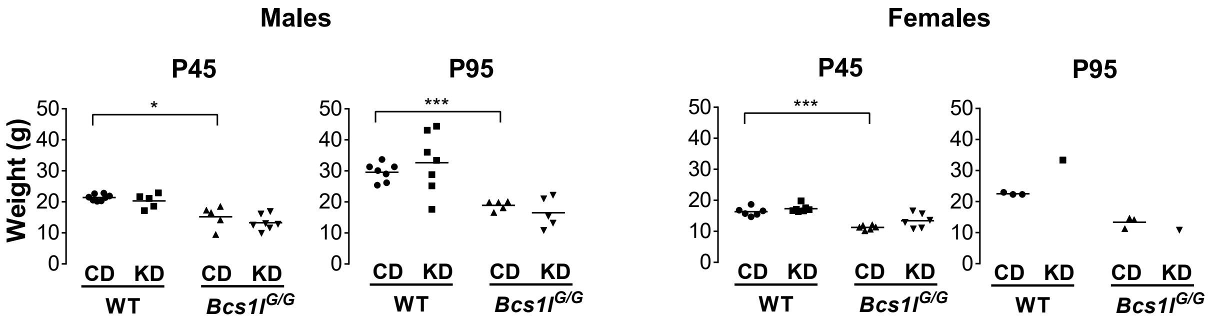

Supplementary Fig. 1. The type of diet did not significantly affect the weight of the animals. The weight of *Bcs1l*$^{G/G}$ mice was approximately 70% of wild-type weight at postnatal day 45 (P45) and 50% at postnatal day 95.

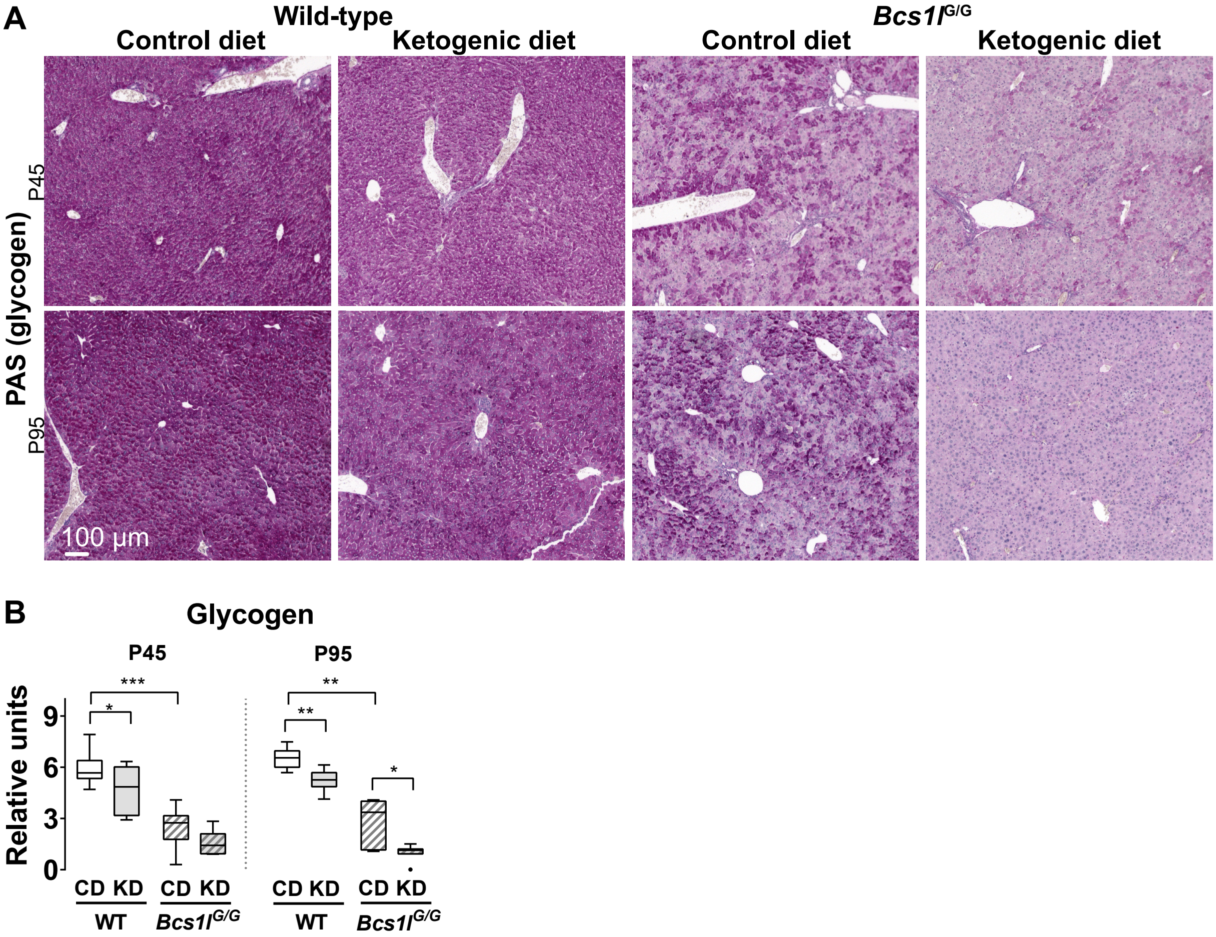

Supplementary Figure 2. In *Bcs1l*$^{G/G}$ mice liver glycogen stores are partially depleted on control diet (CD) and fully depleted on ketogenic diet (KD). (**A**) Representative Periodic Acid-Schiff (PAS) stained liver sections from non-fasted animals. Portal areas lacked glycogen completely in *Bcs1l*$^{G/G}$ mice. Around the central veins some glycogen was observed when the *Bcs1l*$^{G/G}$ mice were on CD. In the wild-type (WT) mice the KD reduced but did not deplete the hepatic glycogen stores. (**B**) Image quantification of liver glycogen content based on intensity difference between PAS stained sections with or without diastase digestion. Sample size is 6-8/group and significances are marked * $p < 0.05$, ** $p < 0.01$ and *** $p < 0.001$.

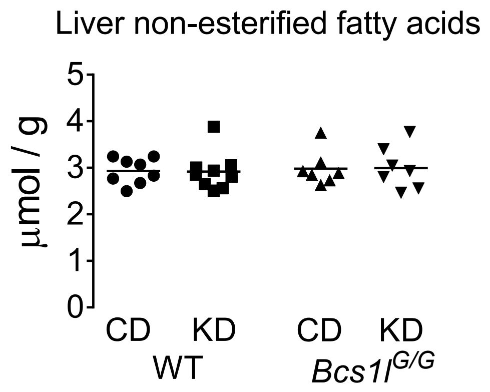

Supplementary Figure 3. Liver non-esterified fatty acids.

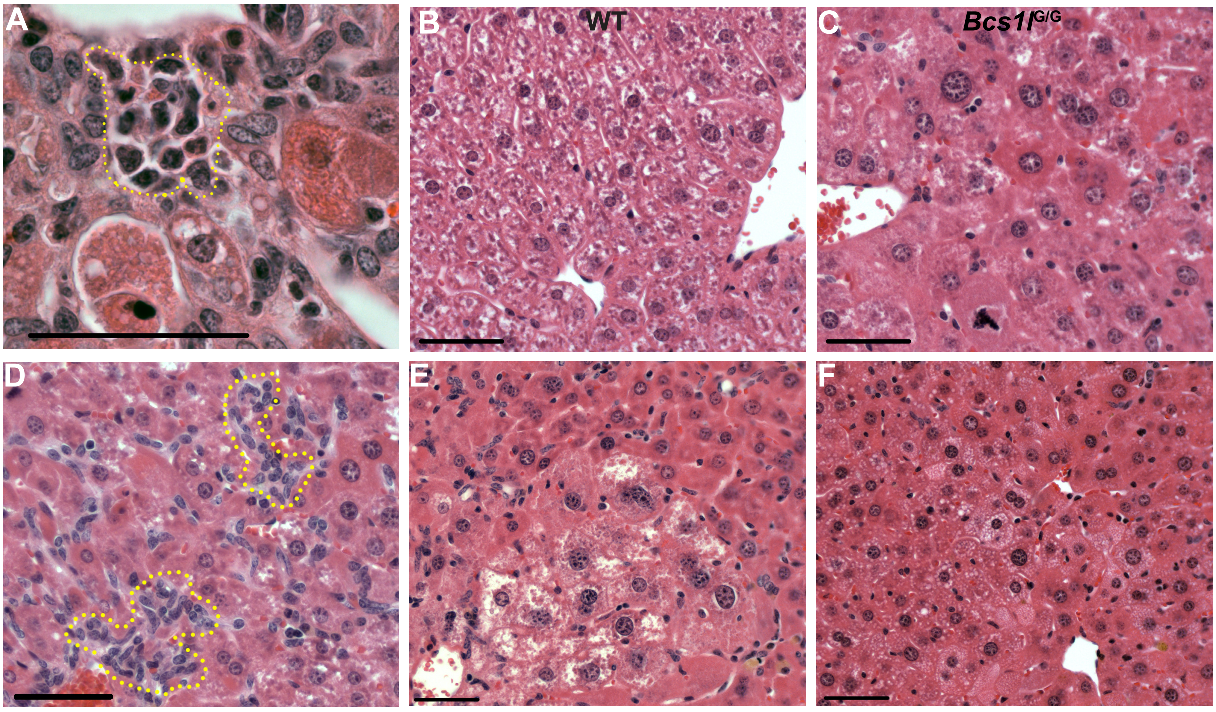

Supplementary Figure 4. High-magnification images of H&E stained liver section showing lesions found in the *Bcs1l*$^{G/G}$ mice. (**A**) A small mixed inflammatory cell infiltrate adjacent to a branch of portal vein in a *Bcs1l*$^{G/G}$ mouse (encircled with yellow dots). The inflammatory cell foci were localized mainly to portal areas. (**B**) A wild-type liver and (**C**) hypertrophied hepatocytes of a *Bcs1l*$^{G/G}$ mouse. Note as a reference, the similar size of the erythrocytes in the lumen of central vein in both sections. The hepatocyte hypertrophy was observed to be panlobular but exacerbated near central veins. (**D**) Hyperplasia of putative hepatic progenitor cells (oval cells). Two clusters are encircled with yellow dots. The lowest cluster is next to a portal triad. The majority of these cells were cytokeratin-7 immunopositive (Fig.2A). (**E**) A focus of cellular alteration in the liver of a *Bcs1l*$^{G/G}$ mouse on control diet. (**F**) A focus of microvesicular lipid accumulation in a liver of ketogenic diet-fed *Bcs1l*$^{G/G}$ mouse. Scale bars, 50 µm

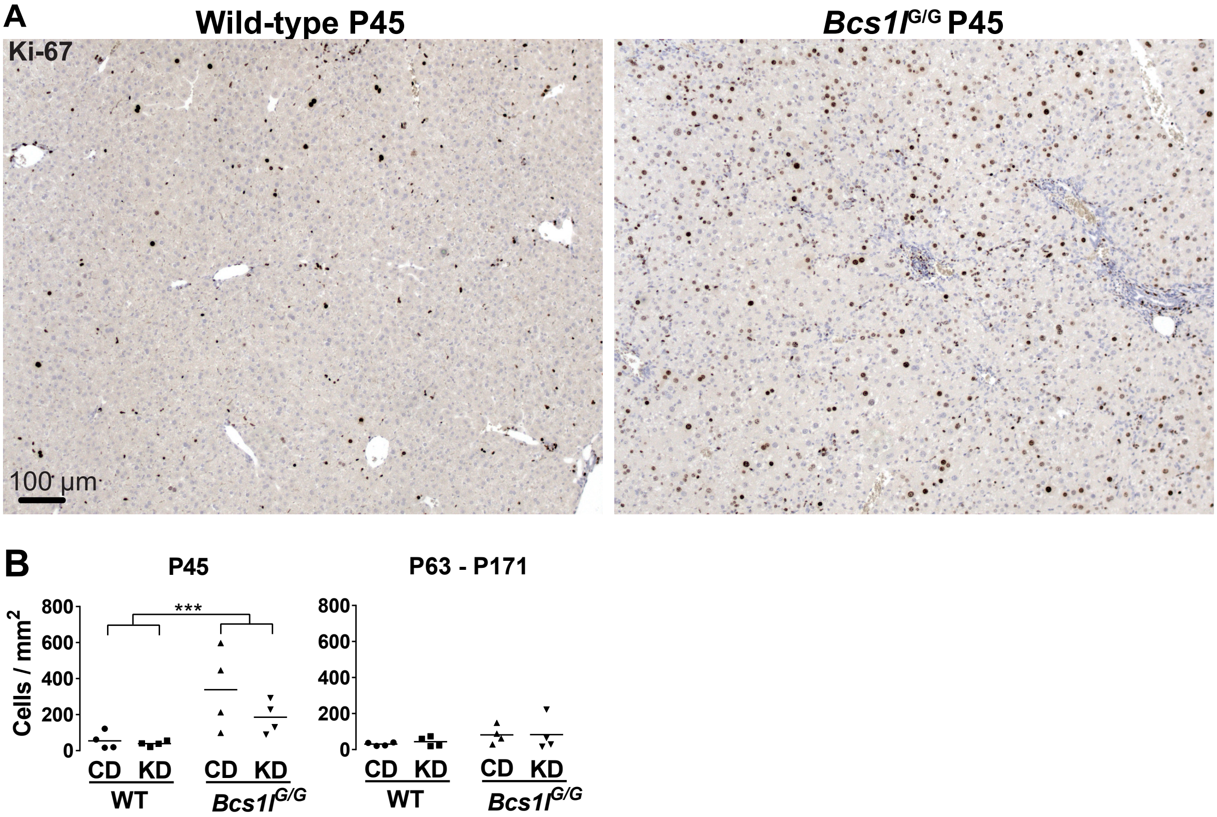

Supplementary Figure 5. Increased mitotic activity is observed in the liver of *Bcs1l*$^{G/G}$ mice at postnatal day 45. (**A**) Representative immunostainings for the proliferation marker Ki-67. (**B**) Quantification of Ki-67 positive cells. In the P63 - P171 graph the samples analyzed are from mice aged between 63 and 171 days. CD, control diet; KD, ketogenic diet; WT, wild-type; ***, $p<0.001$ (t-test)

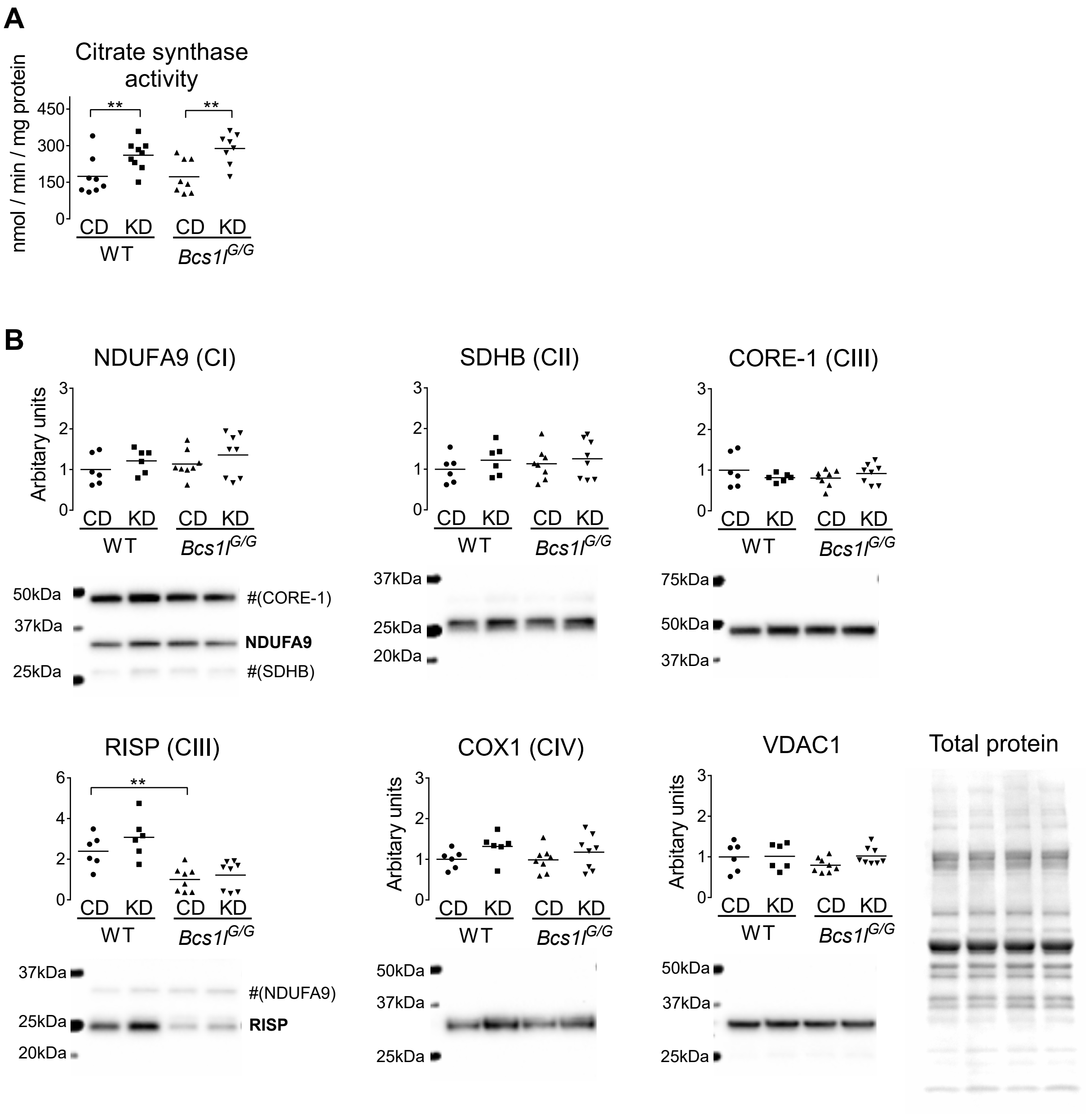

Supplementary Figure 6. Citrate synthase activity and expression of selected mitochondrial proteins in skeletal muscle (quadriceps). (**A**) Citrate synthase activity in tissue lysate normalized to protein concentration. (**B**) Western blot analysis of mitochondrial proteins. Total protein staining of membrane is shown as a control. # The additional bands are remnants from previous detections (marked inside brackets). **, $p<0.01$

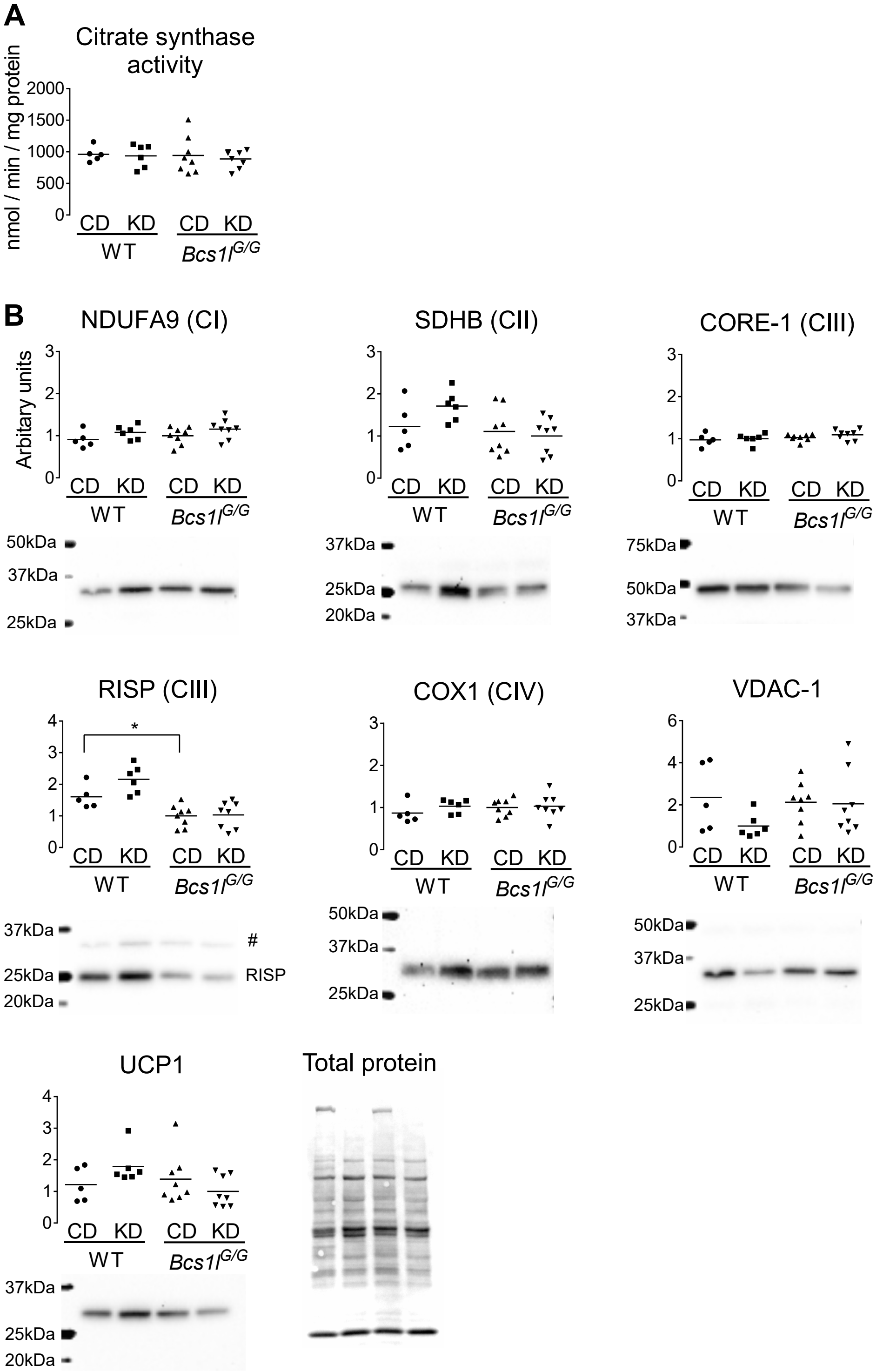

Supplementary Figure 7. Citrate synthase activity and expression of selected mitochondrial proteins in brown adipose tissue. (**A**) Citrate synthase activity in tissue lysate normalized to protein concentration. The results were similar when presented relative to tissue weight. (**B**) Western blot analysis of mitochondrial proteins. Total protein staining of membrane is shown as a control. # The additional bands are remnants from a previous detection (NDUFA9). * p=0.014

Supplementary Table 1. Enriched pathways in trancriptome data, *Bcs1l*$^{G/G}$ on CD compared to WT on CD

Pathway	Overlap	Pathway size	-log$_{10}$[p-value]*	Source
**Up-regulated genes**				
Cytoplasmic Ribosomal Proteins	50	88	46.4	Wikipathways
Metabolism of proteins	92	686	25.0	Reactome
Lysosome - Homo sapiens (human)	24	123	10.0	KEGG
Glutathione metabolism - Homo sapiens (human)	13	51	7.1	KEGG
Mitochondrial protein import	12	54	5.9	Reactome
TCA Cycle	7	17	5.6	Wikipathways
Platelet degranulation	14	82	5.4	Reactome
Y branching of actin filaments	6	17	4.5	BioCarta
Iron uptake and transport	9	43	4.4	Reactome
Validated targets of C-MYC transcriptional activation	13	89	4.3	PID
Aryl Hydrocarbon Receptor	9	46	4.1	Wikipathways
Superpathway of purine nucleotide salvage	10	57	4.1	HumanCyc
MHC class II antigen presentation	10	58	4.0	Reactome
RAC1 signaling pathway	9	54	3.6	PID
Phagosomal maturation (early endosomal stage)	7	33	3.5	Reactome
mtor signaling pathway	6	25	3.4	BioCarta
Insulin receptor recycling	6	26	3.3	Reactome
NRF2 pathway	15	140	3.3	Wikipathways
Fatty acid & beta oxidation	5	19	3.1	HumanCyc
Warburg Effect	7	45	2.7	SMPDB
PPAR Alpha Pathway	5	26	2.5	Wikipathways
HIF-1-alpha transcription factor network	8	66	2.3	PID
DNA Damage Response	8	68	2.2	Wikipathways
Aryl Hydrocarbon Receptor Pathway	6	46	2.0	Wikipathways
**Down-regulated genes**				
Metabolism of amino acids and derivatives	43	150	29.6	Reactome
Complement and coagulation cascades - Homo sapiens (human)	25	69	20.1	KEGG
Peroxisome - Homo sapiens (human)	17	83	9.4	KEGG
Metabolism of lipids and lipoproteins	44	516	9.2	Reactome
L-kynurenine degradation	8	13	9.1	HumanCyc
Urea cycle	5	6	6.8	HumanCyc
Synthesis of bile acids and bile salts via 7alpha-hydroxycholesterol	8	24	6.5	Reactome
NAD *de novo* biosynthesis	6	12	6.2	HumanCyc
PPAR signaling pathway - Homo sapiens (human)	12	69	6.0	KEGG
Gluconeogenesis	6	22	4.4	SMPDB
Drug metabolism - cytochrome P450 - Homo sapiens (human)	9	68	3.7	KEGG
Glycolysis	4	15	3.0	SMPDB
Phase II conjugation	9	95	2.6	Reactome
Peroxisomal lipid metabolism	4	21	2.5	Reactome

Supplementary Table 1 (continued). *Bcs1l* $^{G/G}$ on KD compared to *Bcs1l* $^{G/G}$ on CD

Pathway	Overlap	Pathway size	$-\log_{10}$[p-value]*	Source
**Up-regulated genes**				
Omega-6 fatty acid metabolism	17	28	23.5	EHMN
Valine, leucine and isoleucine degradation - Homo sapiens (human)	20	47	23.5	KEGG
Peroxisome - Homo sapiens (human)	24	83	23.4	KEGG
Saturated fatty acids beta-oxidation	16	25	22.7	EHMN
Mono-unsaturated fatty acid beta-oxidation	12	21	16.2	EHMN
Fatty acid, triacylglycerol, and ketone body metabolism	20	98	16.2	Reactome
Metabolism of amino acids and derivatives	23	150	15.7	Reactome
Leukotriene metabolism	20	104	15.7	EHMN
PPAR signaling pathway - Homo sapiens (human)	13	69	10.2	KEGG
Omega-3 fatty acid metabolism	7	16	8.6	EHMN
Bile acid biosynthesis	10	53	8.0	EHMN
Fatty Acid Biosynthesis	7	22	7.4	Wikipathways
Synthesis of Ketone Bodies	4	4	7.2	Reactome
Urea cycle and metabolism of arginine, proline, glutamate, aspartate and asparagine	10	104	5.2	EHMN
Synthesis of bile acids and bile salts via 7alpha-hydroxycholesterol	5	24	4.5	Reactome
PPAR Alpha Pathway	5	26	4.3	Wikipathways
Pyrimidine nucleotides nucleosides metabolism	4	51	2.0	INOH
**Down-regulated genes**				
Cytoplasmic Ribosomal Proteins	14	88	10.9	Wikipathways
Translation	17	160	10.2	Reactome
Glutathione-mediated detoxification	6	24	6.2	HumanCyc
NRF2 pathway	10	140	4.6	Wikipathways
Lysosome - Homo sapiens (human)	9	123	4.3	KEGG
HIF-1-alpha transcription factor network	6	66	3.5	PID
Validated targets of C-MYC transcriptional activation	6	89	2.9	PID
Iron uptake and transport	4	43	2.6	Reactome
Warburg Effect	4	45	2.5	SMPDB
Aryl Hydrocarbon Receptor Pathway	4	46	2.4	Wikipathways
Metabolism of amino acids and derivatives	7	150	2.3	Reactome
Phagosome - Homo sapiens (human)	7	153	2.3	KEGG
Caspase Cascade in Apoptosis	4	57	2.1	PID
Superpathway of purine nucleotide salvage	4	57	2.1	HumanCyc

Supplementary Table 1 (continued). WT on KD compared to WT on CD

Pathway	Overlap	Pathway size	$-\log_{10}$[p-value]*	Source
**Up-regulated genes**				
Valine, leucine and isoleucine degradation - Homo sapiens (human)	13	47	15.4	KEGG
Mitochondrial Fatty Acid Beta-Oxidation	9	15	14.6	Reactome
Omega-6 fatty acid metabolism	10	28	13.2	EHMN
Fatty acid, triacylglycerol, and ketone body metabolism	14	98	12.3	Reactome
Synthesis of Ketone Bodies	4	4	8.1	Reactome
Tryptophan metabolism - Homo sapiens (human)	7	40	7.0	KEGG
Peroxisome - Homo sapiens (human)	8	83	5.9	KEGG
Citric Acid Cycle	5	21	5.8	SMPDB
PPAR signaling pathway - Homo sapiens (human)	7	69	5.3	KEGG
Warburg Effect	5	45	4.1	SMPDB
Urea cycle and metabolism of arginine, proline, glutamate, aspartate and asparagine	6	104	3.3	EHMN
Lysosome - Homo sapiens (human)	6	123	2.9	KEGG
Xenobiotics metabolism	4	51	2.8	EHMN
Bile acid biosynthesis	4	53	2.8	EHMN
**Down-regulated genes**				
Metabolism of amino acids and derivatives	9	150	5.8	Reactome
Phase II conjugation	7	95	5.2	Reactome
Drug metabolism - cytochrome P450 - Homo sapiens (human)	6	68	5.0	KEGG
Glutathione-mediated detoxification	4	24	4.6	HumanCyc

False discovery rate (Benjamini-Hockenberg) is less than 0.05 for all enrichments. Redundances have been reduced. * Hypergeometric test, Abbreviations: KEGG, Kyoto Encyclopedia of Genes and Genomes; EHMN, Edinburgh Human Metabolic Network; SMPDB, Small Molecule Pathway Database; HumanCyc, Encyclopedia of Human Genes and Metabolism, INOH, Integrating Network Objects with Hierarchie; PID, Pathway Interaction Database

Supplementary Table 2. The most differentially expressed genes, *Bcs1l*$^{G/G}$ on CD compared to WT on CD

Gene	Description	WT: CD < KD<br>Fold Change	WT CD < *Bcs1l*$^{G/G}$ CD<br>Fold Change	*Bcs1l*$^{G/G}$: CD < KD<br>Fold change
Gpnmb	Glycoprotein (transmembrane) nmb	3.0	**278.8**	-2.2
Sprr1a	Small proline-rich protein 1A	-1.7	**120.2**	-2.6
Gsta1	Glutathione S-transferase, alpha 1 (Ya)	-1.2	**86.8**	-4.4
Pdk4	Pyruvate dehydrogenase kinase, isoenzyme 4	-1.3	**56.3**	-2.5
Ly6d	Lymphocyte antigen 6 complex, locus D	-1.0	**47.4**	-2.1
Haus8	4HAUS augmin-like complex, subunit 8	3.9	**46.8**	-2.9
Nupr1	Nuclear protein transcription regulator 1	-2.1	**46.4**	-2.4
Gm3776		-5.6	**40.1**	-8.1
Lgals3	Lectin, galactose binding, soluble 3	1.2	**34.8**	-2.1
Pmm1	Phosphomannomutase 1	2.1	**24.1**	-2.0
Akr1b7	Aldo-keto reductase family 1, member B7	-2.7	**23.5**	-7.7
Cd63	CD63 antigen	-1.5	**20.9**	-2.1
Col1a1	Collagen, type I, alpha 1	2.8	**14.5**	-1.6
Tnfrsf12a	Tumor necrosis factor receptor superfamily, member 12a	-1.6	**12.6**	-2.7
Myc	Myelocytomatosis oncogene	-2.5	**12.3**	-2.7
Ddit4l	DNA-damage-inducible transcript 4-like	1.2	**11.5**	-3.7
Gsta2	Glutathione S-transferase, alpha 2 (Yc2)	1.3	**9.5**	-2.6
Anxa2	Annexin A2	1.5	**9.2**	-1.4
Ddit3	DNA-damage inducible transcript 3	1.2	**9.2**	-2.6
Gstm3	Glutathione S-transferase, mu 3	-4.8	**8.6**	-9.2
Cd68	CD68 antigen	1.2	**8.5**	-2.4
Vcam1	Vascular cell adhesion molecule 1	2.1	**8.4**	-1.3
Slc20a1	Solute carrier family 20, member 1	-1.0	**8.0**	-1.8
S100a11	S100 calcium binding protein A11	1.4	**8.0**	-2.0
Maoa	Monoamine oxidase A	-3.0	**7.9**	-3.4
Gdf15	Growth differentiation factor 15	5.2	**7.8**	-2.6
Lpl	Lipoprotein lipase	1.4	**7.7**	-1.2
Vim	Vimentin	1.8	**7.5**	-2.2
Spp1	Secreted phosphoprotein 1	1.0	**6.8**	-2.7
Krt8	Keratin 8	1.5	**6.7**	-2.2
Ube2c	Ubiquitin-conjugating enzyme E2C	-1.4	**6.7**	-1.9
Pvr	Poliovirus receptor	-1.1	**6.6**	-2.4
Hmox1	Heme oxygenase 1	2.3	**6.6**	-1.8
Ccl6	Chemokine (C-C motif) ligand 6	1.1	**6.5**	-1.2
Kif3a	Kinesin family member 3A	1.9	**6.5**	-1.9
Serpina1e	Serine (or cysteine) peptidase inhibitor, clade A, member 1E	-5.1	**-21.7**	-3.0
Lifr	Leukemia inhibitory factor receptor	-1.9	**-14.9**	-1.1
Mup3	Major urinary protein 3	-1.9	**-12.7**	1.1
Ces3b	Carboxylesterase 3B	-2.0	**-11.7**	-1.5
Mup7	Major urinary protein 7	-1.5	**-11.7**	1.6
Cyp2f2	Cytochrome P450, family 2, subfamily f, polypeptide 2	-1.6	**-9.9**	2.1
Sult2a8	Sulfotransferase family 2A, dehydroepiandrosterone (DHEA)-preferring, member 8	2.0	**-9.5**	8.1
Dio1	Deiodinase, iodothyronine, type I	1.3	**-8.2**	4.6
Slco1a1	Solute carrier organic anion transporter family, member 1a1	-1.9	**-7.2**	-4.7

Supplementary Table 2 (continued). *Bcs1l*$^{G/G}$ on KD compared to *Bcs1l*$^{G/G}$ on CD

Gene	Description	WT: CD < KD<br>Fold Change	WT CD < *Bcs1l*$^{G/G}$ CD<br>Fold Change	*Bcs1l*$^{G/G}$: CD < KD<br>Fold change
Sult2a8	Sulfotransferase family 2A, dehydroepiandrosterone (DHEA)-preferring, member 8	2.0	-9.5	**8.1**
Sult2a2	Sulfotransferase family 2A, dehydroepiandrosterone (DHEA)-preferring, member 2	4.3	1.0	**7.4**
Fabp2	Fatty acid binding protein 2, intestinal	2.5	-1.5	**6.4**
Vnn1	Vanin 1	3.9	-1.7	**6.1**
Ugt3a1	UDP glycosyltransferases 3 family, polypeptide A1	1.8	-6.1	**5.9**
Rgs16	Regulator of G-protein signaling 16	3.9	-4.3	**5.6**
Sult2a1	Sulfotransferase family 2A, dehydroepiandrosterone (DHEA)-preferring, member 1	3.8	1.3	**4.7**
Dio1	Deiodinase, iodothyronine, type I	1.3	-8.2	**4.6**
Tmem134	Transmembrane protein 134	4.5	-1.1	**4.3**
Retsat	Retinol saturase (all trans retinol 13,14 reductase)	1.8	-1.4	**4.0**
Tpm2	Tropomyosin 2, beta	2.7	1.0	**3.8**
Cyp4a32	Cytochrome P450, family 4, subfamily a, polypeptide 32	3.7	1.4	**3.8**
Uqcrb	Ubiquinol-cytochrome c reductase binding protein	3.6	1.6	**3.7**
Ces1f	Carboxylesterase 1F	1.3	-3.7	**3.4**
Acaa1b	Acetyl-Coenzyme A acyltransferase 1B	2.5	-1.1	**3.3**
Sugct	Succinyl-CoA glutarate-CoA transferase	1.6	-1.9	**3.1**
Aldh3a2	Aldehyde dehydrogenase family 3, subfamily A2	4.0	1.5	**3.1**
Cyp8b1	Cytochrome P450, family 8, subfamily b, polypeptide 1	-1.0	-3.1	**2.9**
Cyp4a10	Cytochrome P450, family 4, subfamily a, polypeptide 10	3.1	1.9	**2.9**
Ehhadh	Enoyl-Coenzyme A, hydratase/3-hydroxyacyl Coenzyme A dehydrogenase	2.6	2.9	**2.8**
Acat1	Acetyl-Coenzyme A acetyltransferase 1	1.8	-1.8	**2.8**
Scd1	Stearoyl-Coenzyme A desaturase 1	-66.6	-1.3	**-60.2**
Tff3	Trefoil factor 3, intestinal	-37.9	1.2	**-12.1**
Gstm3	Glutathione S-transferase, mu 3	-4.8	8.6	**-9.2**
Gm3776		-5.6	40.1	**-8.1**
Akr1b7	Aldo-keto reductase family 1, member B7	-2.7	23.5	**-7.7**
Acmsd	Amino carboxymuconate semialdehyde decarboxylase	-4.1	1.5	**-6.8**
H2-Q1	Histocompatibility 2, Q region locus 1	-3.1	5.5	**-4.9**
Slco1a1	Solute carrier organic anion transporter family, member 1a1	-1.9	-7.2	**-4.7**
Cps1	Carbamoyl-phosphate synthetase 1	-1.2	-5.8	**-4.5**
Gsta1	Glutathione S-transferase, alpha 1 (Ya)	-1.2	86.8	**-4.4**
Btc	Betacellulin, epidermal growth factor family member	-1.1	6.2	**-3.9**
Gstm2	Glutathione S-transferase, mu 2	-2.6	2.4	**-3.7**
Ddit4l	DNA-damage-inducible transcript 4-like	1.2	11.5	**-3.7**
Nnmt	Nicotinamide N-methyltransferase	-4.1	-1.1	**-3.5**
Maoa	Monoamine oxidase A	-3.0	7.9	**-3.4**
Hamp	Hepcidin antimicrobial peptide	-2.5	1.3	**-3.2**
Serpina1e	Serine (or cysteine) peptidase inhibitor, clade A, member 1E	-5.1	-21.7	**-3.0**
Haus8	4HAUS augmin-like complex, subunit 8	3.9	46.8	**-2.9**
Igfbp1	Insulin-like growth factor binding protein 1	-1.0	4.4	**-2.9**
Spp1	Secreted phosphoprotein 1	1.0	6.8	**-2.7**
Zfos1	Zinc finger, NFX1-type containing 1, opposite strand RNA 1	1.2	3.5	**-2.7**
Tnfrsf12a	Tumor necrosis factor receptor superfamily, member 12a	-1.6	12.6	**-2.7**
Myc	Myelocytomatosis oncogene	-2.5	12.3	**-2.7**

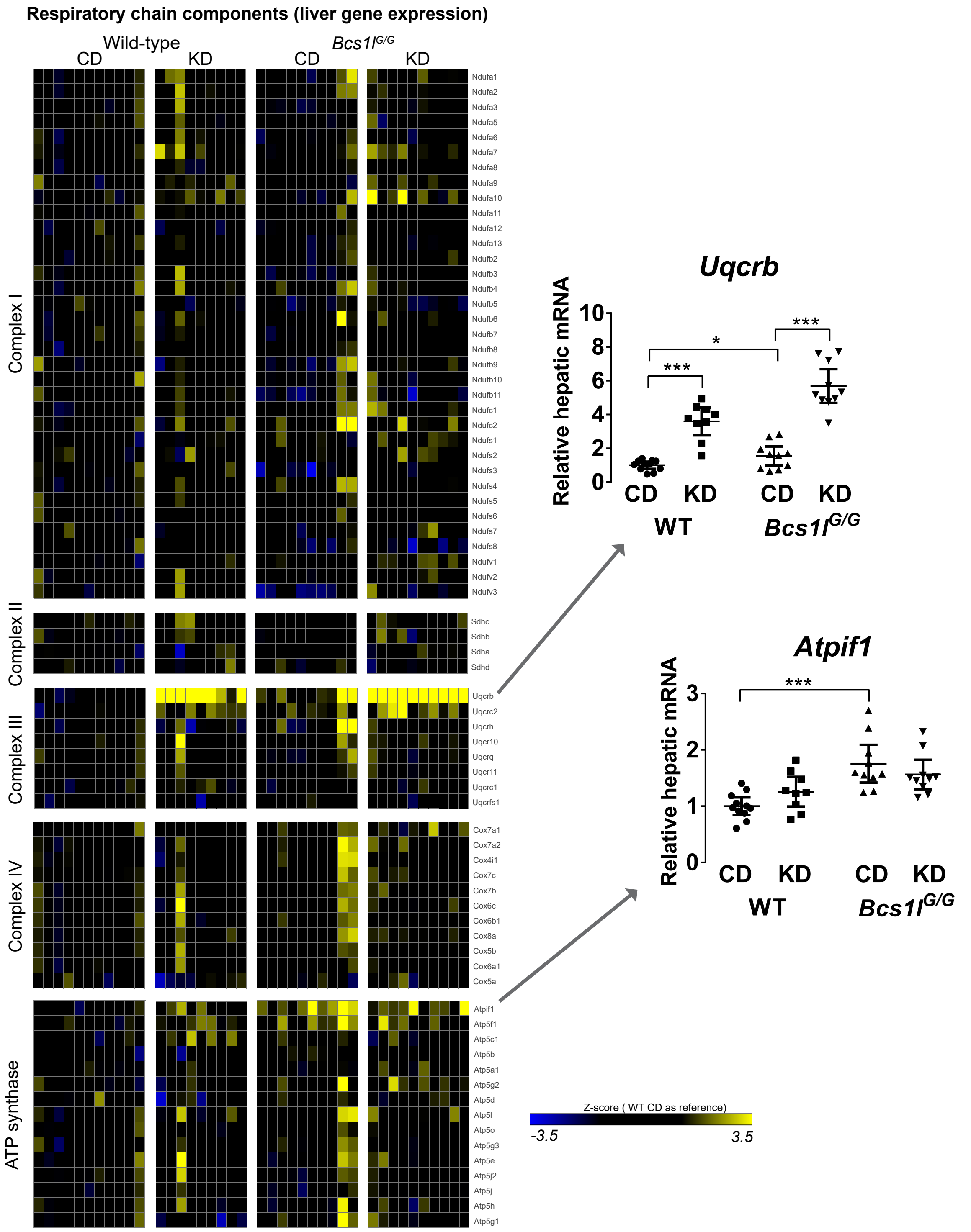

Supplementary Figure 8. Expression of genes encoding respiratory chain components.

* $p<0.05$, *** $p<0.001$

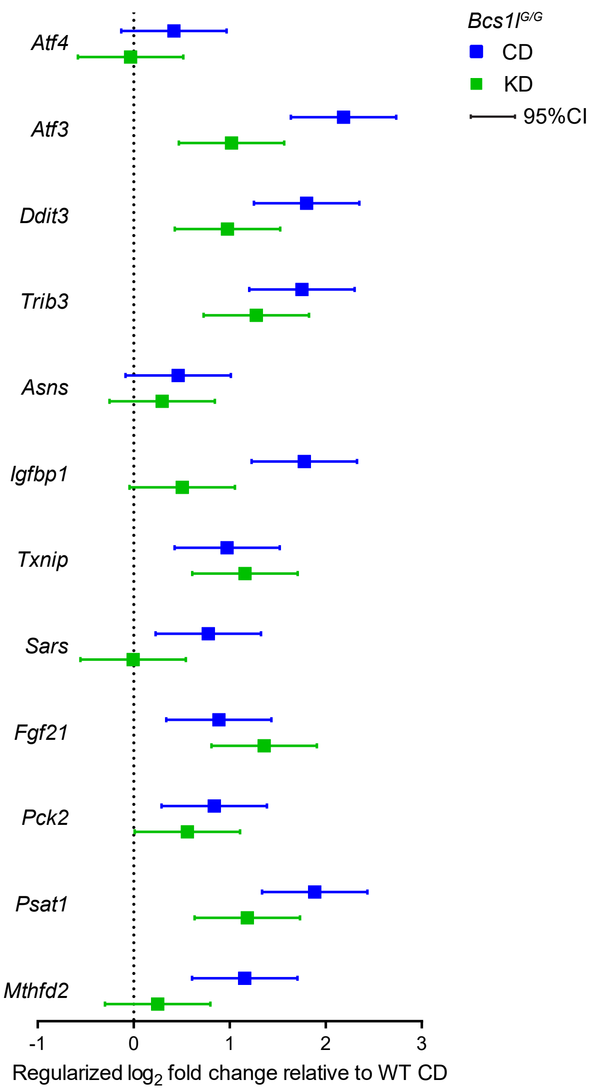

Supplementary Figure 9. Liver gene expression of amino acid starvation response genes[7]. n=9-11/group, the error bars present 95% confidence interval (95%CI). Due to low read count for some transcripts in RNA sequencing, regularized log_2-like transformed data was used as described in DESeq2 vignette[4]. This transformation takes into account the uncertainty associated with low read counts and thus the fold changes are lower than in conventional log_2-scale.

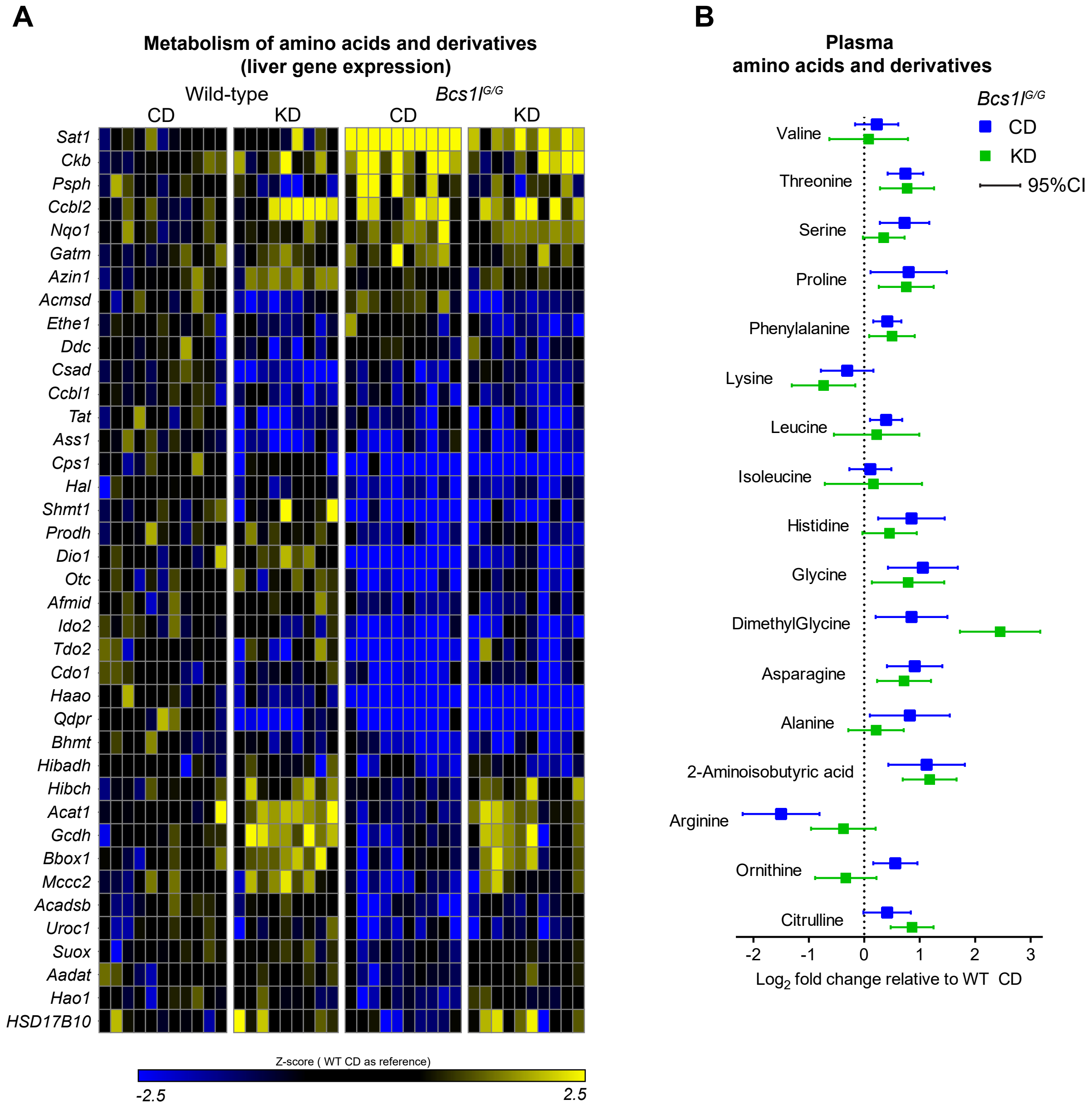

Supplementary Figure 10. Liver transcriptomics and plasma metabolomics reveal altered amino acid metabolism and aminoacidemia in *Bcs1l*$^{G/G}$ mice. (**A**) Differentially expressed genes overlapping with "Metabolism of amino acids and derivatives" pathway from the Reactome database. The 39 most differentially expressed genes are shown from total overlap of 62/150. The expression data is from 45-day old mice. (**B**) Relative concentration of amino acids and derivatives in the plasma of the 95-day old mice. For clarity WT on KD group is not show as only marginal differences were observed compared to WT on CD. The error bars present 95% confidence interval (95%CI). n=6-7/group.

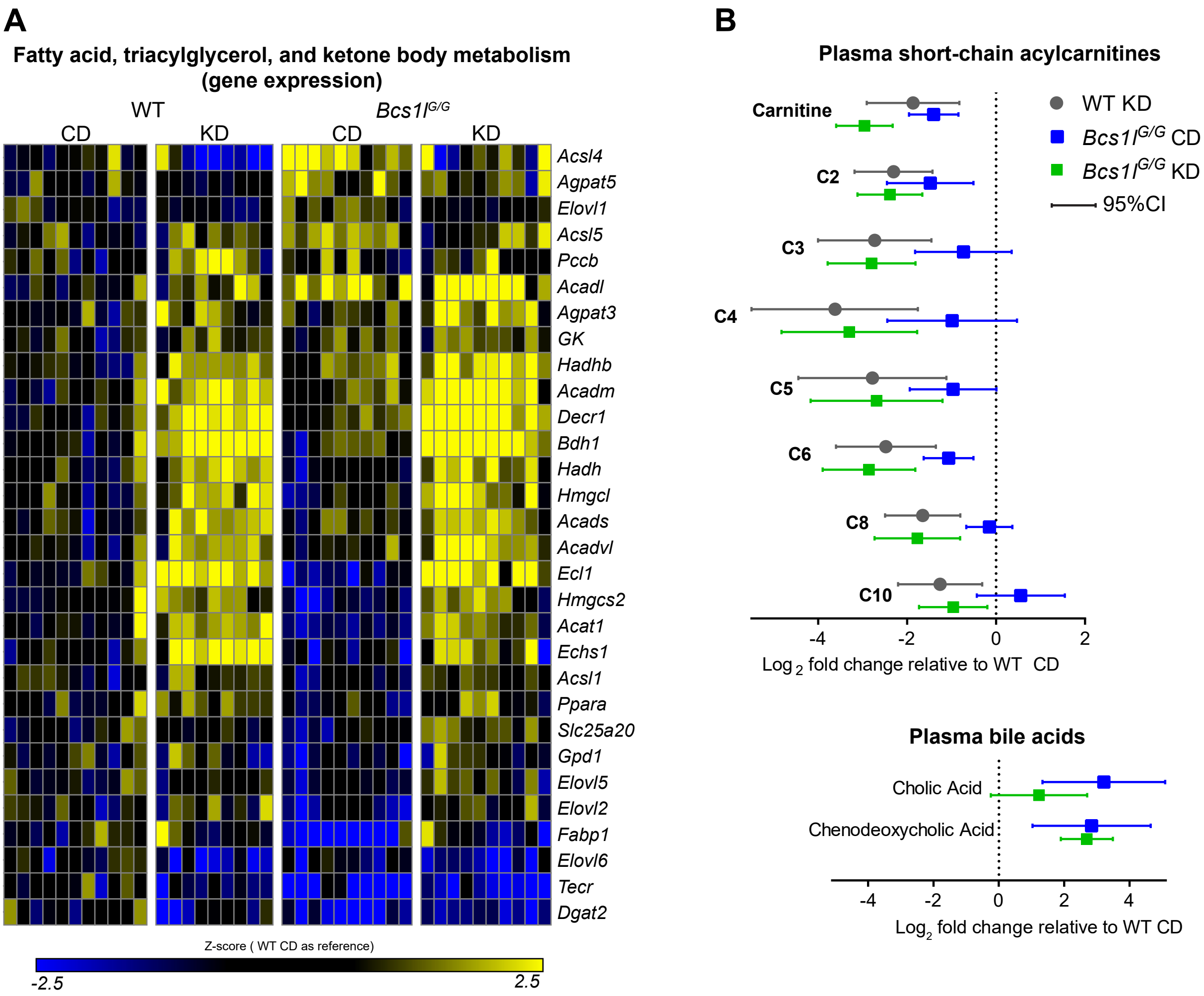

Supplementary Figure 11. Perturbations in lipid metabolism by the *Bcs1l*$^{G/G}$ mutation and KD as shown by liver transcriptome (A) and plasma metabolome (B). The presented genes involved in lipid metabolism are differentially expressed genes which overlap with ”Fatty acid, triacylglycerol, and ketone body metabolism” pathway from the Reactome database. The error bars present 95% confidence interval (95%CI). n=6-7 / group for metabolite analyses.

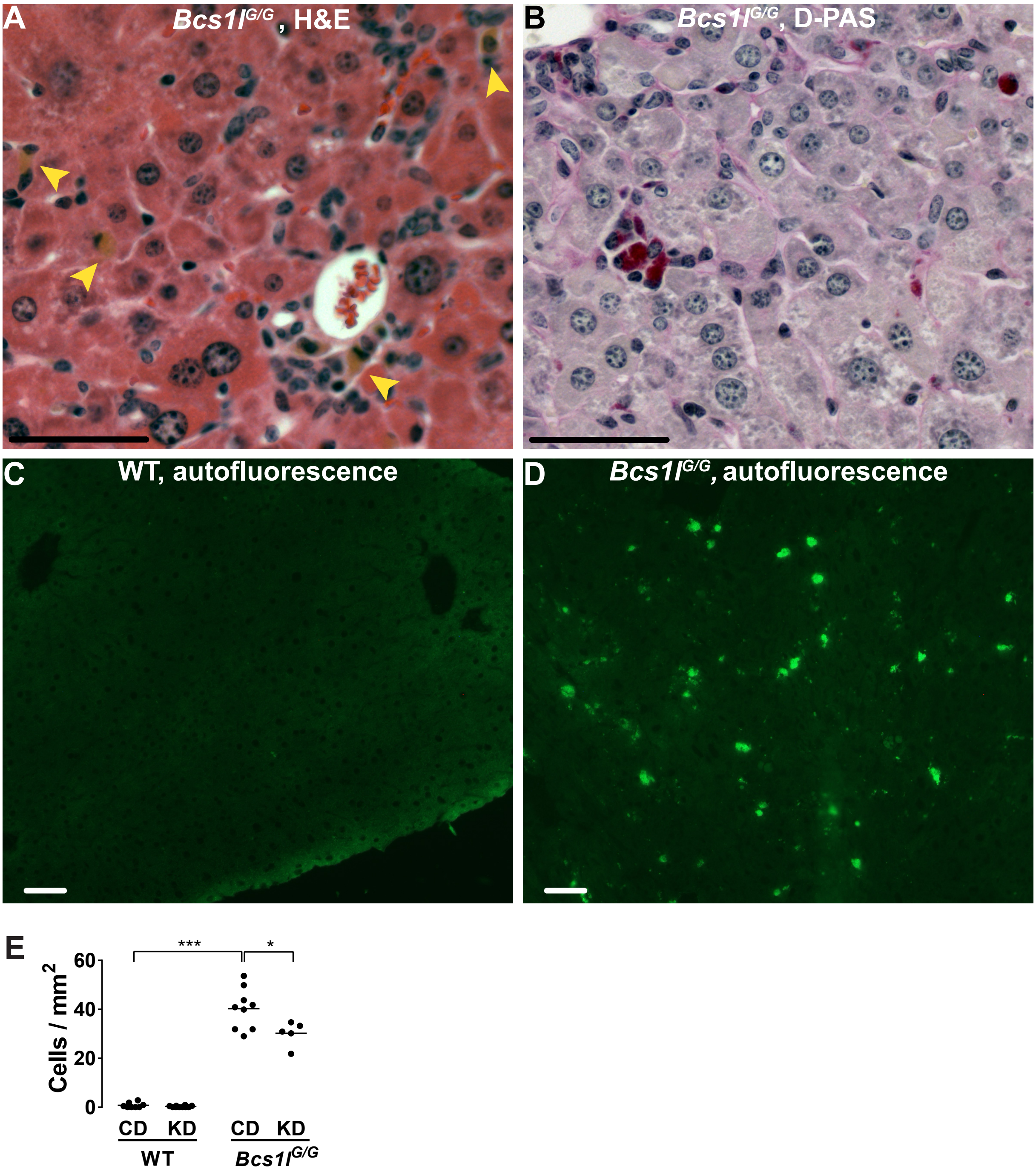

Supplementary Figure 12. Ceroid-lipofuscin -like material accumulates into the liver macrophages of *Bcs1l*$^{G/G}$ mice. (**A**) Macrophage resembling cells with diffuse brownish cytoplasm were found in H&E stained liver sections of *Bcs1l*$^{G/G}$ mice (arrowheads). (**B**) These cells were PAS (Periodic Acid-Schiff) positive in diastase digested liver sections (D-PAS) and (**C-D**) were autofluorescent. (**C-D**) Autofluorescence of unstained liver cryosections under 467 - 498 nm excitation and filtered for 513 - 556 nm emission. The autofluorescence extended to yellow-orange spectrum of light (not shown). (**E**) Cell count for D-PAS positive cell. All data shown are from postnatal day 95 time point. A few sporadic ceroid/lipofuscin positive cells could be detected already at postnatal day 45. CD, control diet; KD, ketogenic diet; WT, wild-type; *, $p < 0{,}05$; *** $p < 0.001$; scale bars, 50 µm

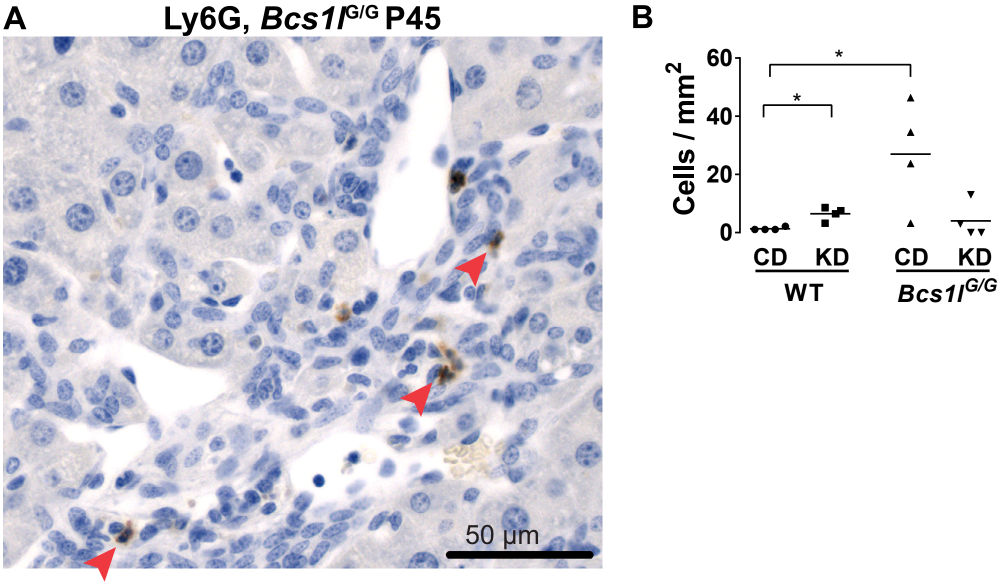

Supplementary Figure 13. The number of neutrophils is increased in the liver of *Bcs1l*$^{G/G}$ mice and wild-type mice on ketogenic diet. (**A**) A representative immunostaining against Ly6G antigen. The arrows point to the neutrophils. The neutrophils did not form inflammatory clusters. (**B**) Count of positive cells. P45, postnatal day 45; CD, control diet; KD, ketogenic diet; WT, wild-type; *, $p<0.05$ (Kruskal-Wallis test followed by pair-wise comparisons with Mann-Whitney U tests)

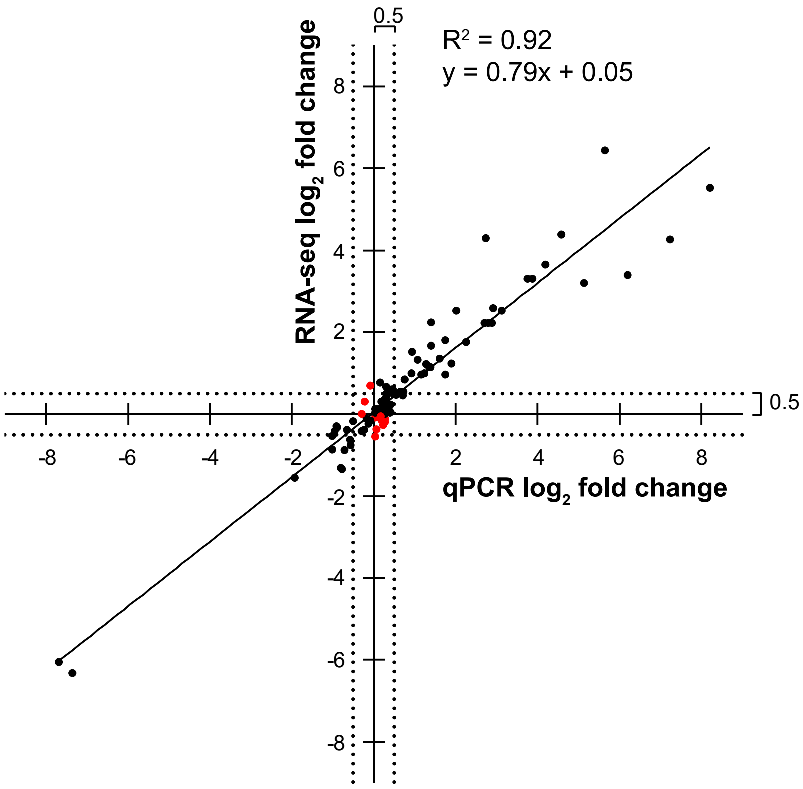

Supplementary Figure 14. Correlation between RNA-seq and qPCR. In total the expression of 45 genes was validated by qPCR. Genes with less than eight read counts in RNA-seq were excluded from the comparison. Red dots show opposing effect by the two methods. In general, high agreement was found between the two methods for genes that were within dynamic range of RNAseq and had absolute fold change higher than 1.4 (0.5 in $\log_2$ scale).

.

Supplementary Table 3. Antibodies used in immunohistochemistry (IHC) and Western blotting (WB)

Antibody	Antigen	Host	Application	Pretreatment	Clone	Cat. no.	Manufacturer
CK7	cytokeratin-7	rabbit	IHC	HIER		15539-1-AP	ProteinTech Inc.
Ki67	MKI67	rabbit	IHC	HIER	SP6	ab16667	Abcam Ltd.
α-SMA	alpha smooth muscle actin	mouse	IHC	-	1A4	A5691	Sigma-Aldrich
F4/80	EMR1	rat	IHC	PIER	CI:A3-1	MCA497GA	Bio-Rad
IBA1	AIF1	rabbit	IHC	-	-	019-19741	Wako Ltd.
NIMP-R14	Ly6G	rat	IHC	HIER	NIMP-R14	sc-59338	Santa Cruz Biotechnology
NDUFA9	NDUFA9 (CI subunit)	mouse	WB	-	20C11B11B11	ab14713	Abcam Ltd.
SDHB	SDHB (CII subunit)	mouse	WB	-	21A11AE7	ab14714	Abcam Ltd.
UQCRFS1	UQCRFS1, RISP (CIII subunit)	mouse	WB	-	5A5	ab14746	Abcam Ltd.
UQCRC1	UQCRC1, CORE-1 (CIII subunit)	mouse	WB		16D10AD9AH5	ab110252	Abcam Ltd.
MTCO1	MTCO1, COX1 (CIV subunit)	mouse	WB	-	1D6E1A8	ab14705	Abcam Ltd.
VDAC1	VDAC1	rabbit	WB	-	EPR10852(B)	ab154856	Abcam Ltd.
UCP1	UCP1	rabbit	WB	-	-	ab10983	Abcam Ltd.

Abbreviations: HIER, heat-induced antigen retrieval (boiling in 10mM sodium citrate, 0.05% Tween-20, pH 6.0 for 10-20 minutes), PIER, Proteolytic Induced Epitope Retrieval (Protenase K 20 µg/mL, 5 min +37°C)

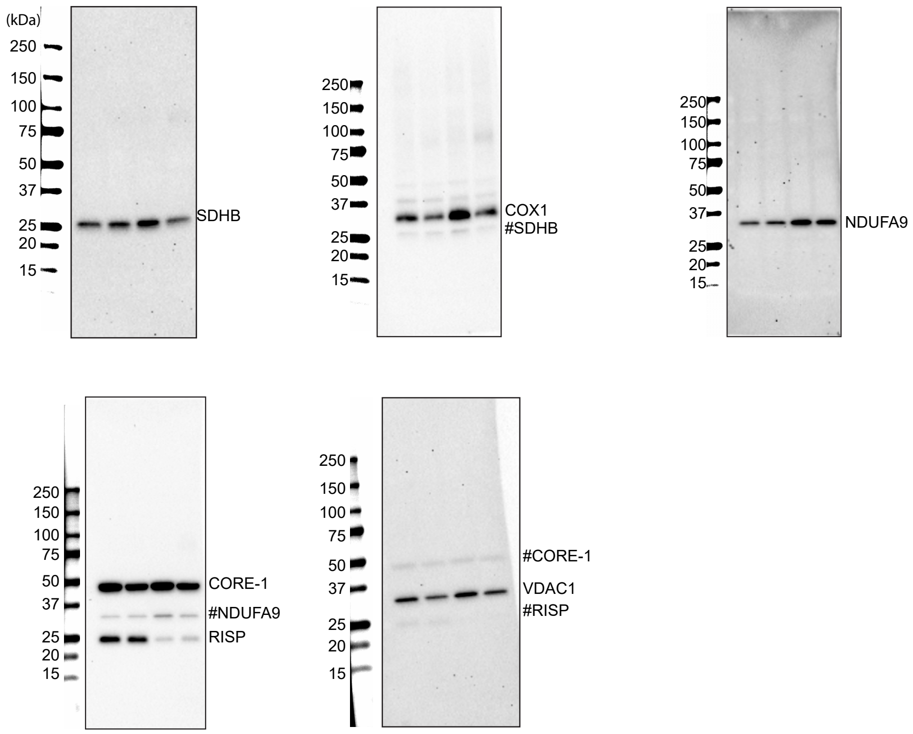

Supplementary Fig. 15. Full-length immunoblots related to Fig. 4C. Additional bands and their explanation are marked with #. Precision Plus Protein All Blue standard (Bio-Rad) was used to estimate size of the proteins.

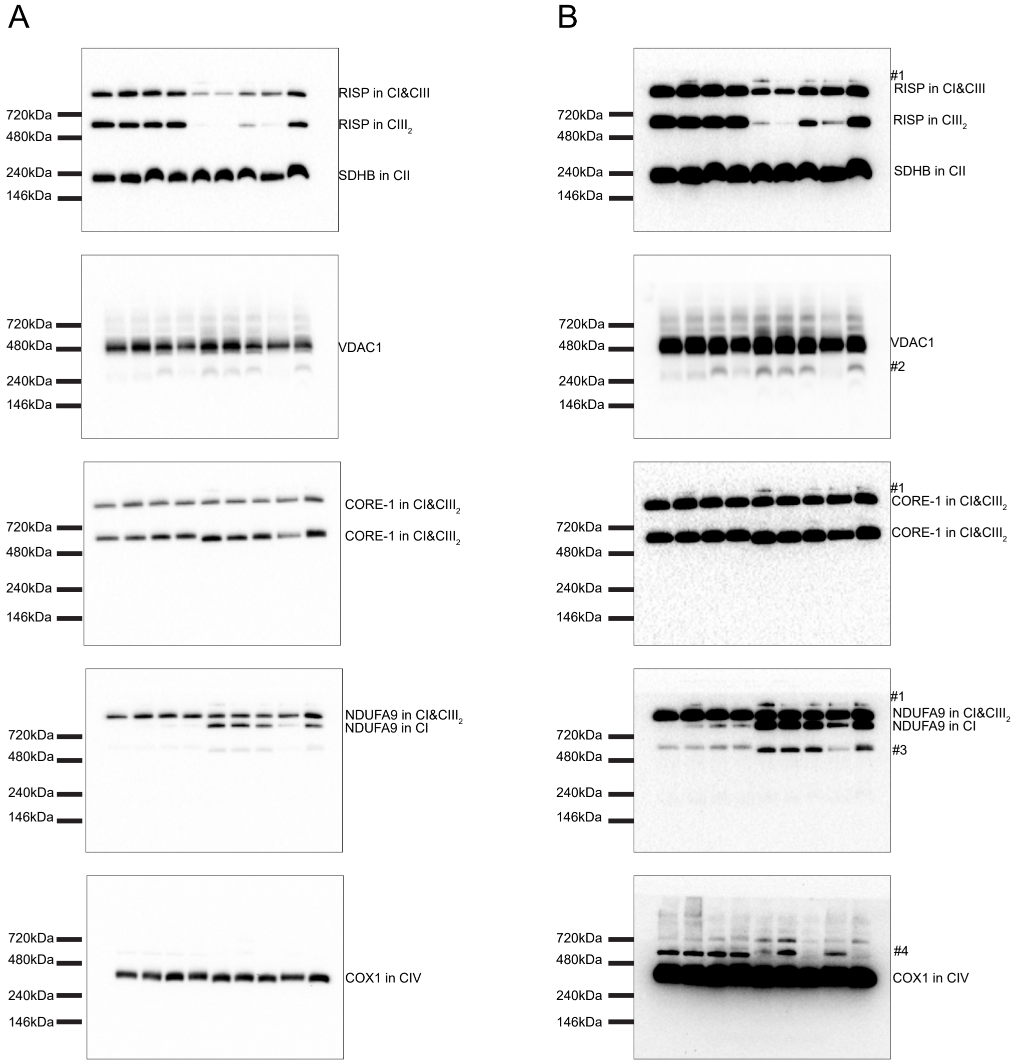

Supplementary Fig. 16. Full-length images of immunodetections used to stain respiratory chain complexes after Blue native gel electrophoresis and western blot (Fig.4E). (**A**) Exposure time set to below saturation of imaging device. (**B**) Overexposed counterparts. NativeMark Unstained Protein Standard (Invitrogen, P/N57030) was used to estimate size of the protein complexes. All images are from the same blot. The last band, present here but not in Fig.4E, is a pooled sample that was used to control blot to blot variation in quantification. #1, Minor bands in supercomplex region (>1000kDa); #2 Residual horseradish peroxidase activity from the previous detection (SDHB); #3 Unstripped CORE-1; #4 unidentified minor bands.
